# A generalization of t-SNE and UMAP to single-cell multimodal omics

**DOI:** 10.1186/s13059-021-02356-5

**Published:** 2021-05-03

**Authors:** Van Hoan Do, Stefan Canzar

**Affiliations:** Gene Center, Ludwig-Maximilians-Universität München, Feodor-Lynen-Str. 25, Munich, Germany

**Keywords:** Data visualization, Single-cell sequencing, Multimodal omics, t-SNE, UMAP, RNA velocity, Protein velocity

## Abstract

Emerging single-cell technologies profile multiple types of molecules within individual cells. A fundamental step in the analysis of the produced high-dimensional data is their visualization using dimensionality reduction techniques such as t-SNE and UMAP. We introduce j-SNE and j-UMAP as their natural generalizations to the joint visualization of multimodal omics data. Our approach automatically learns the relative contribution of each modality to a concise representation of cellular identity that promotes discriminative features but suppresses noise. On eight datasets, j-SNE and j-UMAP produce unified embeddings that better agree with known cell types and that harmonize RNA and protein velocity landscapes.

**Supplementary Information:**

The online version contains supplementary material available at (10.1186/s13059-021-02356-5).

## Background

**Fig. 1:**
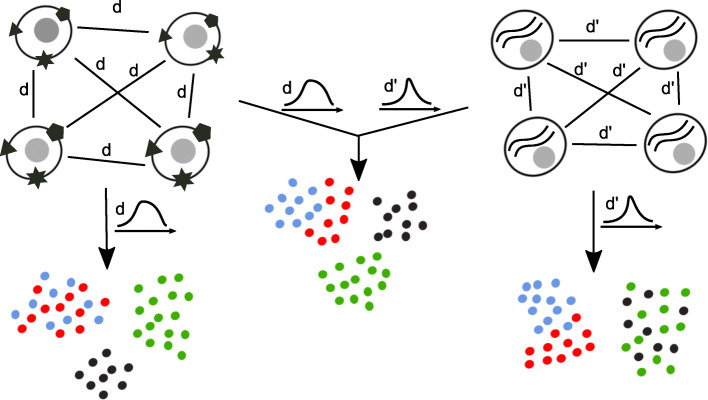
Overview of the joint embedding in JVis. Metrics *d* (left) and *d*^′^ (right) measure the dissimilarity of different cellular phenotypes of individual cells, such as the expression of surface proteins (left) and mRNA (right). t-SNE and UMAP learn a low-dimensional embedding of cells that preserves the distribution of similarities that are quantified based on *d* or *d*^′^ alone, which renders certain cell types indistinguishable to either modality. In this example, blue and red cells cannot be distinguished based on their measured surface proteins, and green and black cells overlap in transcriptomic space. In JVis we generalize t-SNE and UMAP to learn a joint embedding that preserves similarities in all modalities at the same time. We integrate *d* and *d*^′^ in a convex combination of KL divergences (j-SNE) or cross entropies (j-UMAP) between corresponding similarities in low and high-dimensional space. An arrangement of cells that minimizes this convex combination with simultaneously learned weights takes into account similarities and differences in both mRNA and surface protein expression to more accurately represent cellular identity (middle)

Single-cell RNA sequencing has enabled gene expression profiling at single-cell resolution and provided novel opportunities to study cellular heterogeneity, cellular differentiation and development. Emerging single-cell technologies assay multiple modalities such as transcriptome, genome, epigenome, and proteome at the same time [1–3]. The joint analysis of multiple modalities has allowed to resolve subpopulations of cells at higher resolution [4, 5], has helped to infer the “acceleration” of RNA dynamics [6] and to extend time periods over which cell states can be predicted [7], and has linked dynamic changes in chromatin accessibiliy to transcription during cell-fate determination [8]. A fundamental step in the analysis of high-dimensional single-cell data is their visualization in two dimensions. Arguably the most widely used nonlinear dimensionality reduction techniques are t-distributed stochastic neighbor embedding (t-SNE) [9] and uniform manifold approximation and projection (UMAP) [10]. Currently, these techniques are applied to each modality one at a time [1, 8, 11], and separate views of the data need to be reconciled by manual inspection. Here, we generalize t-SNE and UMAP to the joint visualization of multimodal single-cell measurements. While t-SNE and UMAP seek a low-dimensional embedding of cells that preserves similarities in the original (e.g., gene expression) space as well as possible, we propose j-SNE and j-UMAP that simultaneously preserve similarities across all modalities (Fig. 1). Through Python package JVis, they will allow to combine different views of the data into a unified embedding that can help to uncover previously hidden relationships among them. At the same time, our joint embedding schemes learn the relative importance of each modality from the data to reveal a concise representation of cellular identity.

## Results and discussion

In j-SNE, we want to learn a joint embedding $\mathcal {E}$ of cells for each of which we have measured multiple modalities. Analog to t-SNE [9], we want to arrange cells in low-dimensional space such that similarities observed between points in high-dimensional space are preserved, but in all modalities at the same time. Generalizing the objective of t-SNE, we aim to minimize the convex combination of KL divergences of similarities in the original high-dimensional (distribution *P*) and similarities in the embedding low-dimensional space (distribution *Q*) for each modality *k*:

1 $$ C(\mathcal E) = \sum\limits_{k} \alpha_{k} KL\left(P^{(k)}||Q\right) + \lambda \sum\limits_{k} \alpha_{k} \log \alpha_{k},  $$

where coefficients *α* of the convex combination represent the importance of individual modalities towards the final location of points in the embedding. We add a regularization term (with regularization parameter *λ*) that prevents the joint embedding from being biased towards individual modalities. In j-UMAP, we generalize UMAP to multimodal data analogously, minimizing a convex combination of cross entropies instead of KL divergences. We jointly optimize the location of points in the embedding and the importance coefficients *α* of modalities through an alternating optimization scheme: We fix coefficients *α* and find the best point locations by gradient descent, and in turn find optimal coefficients *α* for fixed locations by solving a convex optimization problem. Our approach is described in detail in Additional file 1: Supplementary Methods.

**Fig. 2:**
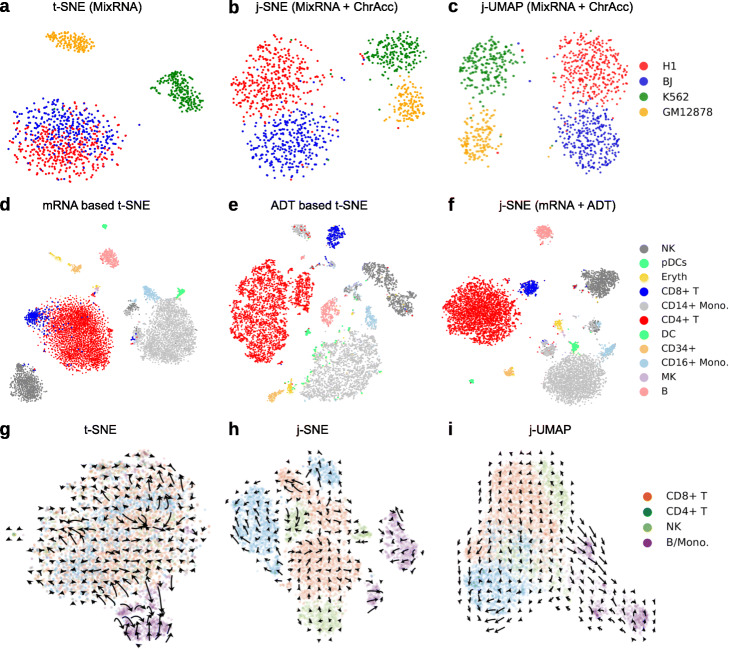
Comparison of cell types and protein acceleration in unimodal and multimodal embeddings. *First row:* Visualization of perturbed SNARE-seq measurements. Accessible chromatin (ChrAcc) and gene expression was measured simultaneously in single cell from human cell lines BJ, H1, K562, and GM12878. Gene expression measurements were randomly shuffled between cell lines BJ and H1 (MixRNA). **a** Conventional t-SNE embedding of cells based on shuffled gene expression alone. **b** j-SNE visualization of shuffled gene expression and (unchanged) chromatin accessibility. **c** j-UMAP visualization of shuffled gene expression and (unchanged) chromatin accessibility. *Second row:* t-SNE/j-SNE visualizations of CBM cells. Cluster labels were identified by Specter. Embeddings were computed from RNA measurements alone (**d**), protein expression (ADT) alone (**e**), or jointly from both (**f**). *Third row:* Protein acceleration in ECCITE-seq (ctrl) data set projected into transcriptom-based t-SNE (**g**), and joint mRNA and surface protein based embeddings j-SNE (**h**), and j-UMAP (**i**)

As proof of concept, we first demonstrate the ability of JVis to integrate modalities with different signal strengths. scRNA-seq, for example, often allows a finer mapping of cell states than single-cell ATAC-seq [12]. We used JVis to compute a joint embedding of accessible chromatin and gene expression measured simultaneously by SNARE-seq [11] in 1047 single cells from cultured human cell lines BJ, H1, K562, and GM12878. Similar to the conventional t-SNE and UMAP embeddings of transcriptomes or chromatin state alone, our joint j-SNE and j-UMAP embeddings clearly separate cells into four distinct clusters (Additional file 1: Fig. S1). Even when randomly shuffling gene expression measurements between cell lines BJ and H1 in a toy experiment, JVis employs chromatin accessibility to disentangle mixed mRNA measurements and separate all four cell lines (Fig. 2a–c and Additional file 1: Fig. S2).

To examine the effectiveness of the joint optimization scheme underlying JVis, we devise a simulation study following a similar strategy as [13]. We used Splatter [14] to simulate joint gene and ADT counts based on model parameters estimated from a real CITE-seq data set [15] in which mRNA and surface protein (ADT) expression were measured in human peripheral blood mononuclear cells (PBMC). We added a third modality that is obtained from gene expression measurements that are randomly shuffled between a random subset of cells. We generated eight synthetic multimodal data sets that vary in the relative abundance of (five) cell types, number of cells, and in the number of genes (Additional file 1: Table S1). In contrast to its conventional counterparts, j-SNE and j-UMAP learn weights for each modality from the data that reflect their relevance to the final embedding. Additional file 1: Figs. S3 and S4 show that these weights distinguish informative from noisy modalities. With an increasing amount of perturbation of the third modality, i.e., an increasing number of cells with shuffled gene expression, JVis assigns a lower weight to the corresponding modality. The rate of weight decrease (and the simultaneous increase mostly in ADT weight) is higher for data sets with a larger number of cells and, as expected, depends on the regularization coefficient *λ*. For *λ* close to 0, weights essentially include a single most informative modality (here ADT, see Additional file 1: Table S2) (Additional file 1: Fig. S5 and Supplementary Methods). Higher penalties associated with non-uniform weights result in a weaker adjustment of weights by the joint optimization scheme. The absolute adjustment of weights associated with cross entropy terms in j-UMAP is less pronounced than the adjustment of weights associated with KL divergences in j-SNE. Finally, in Additional file 1: Fig. S6 we show that weights computed by JVis are robust with respect the precise subset of cells sampled in an experiment.

We measure the positive effect the learned weights have on the accuracy of the final embedding using the Silhouette score [16] that measures how well separated cell types are in the embedding, and metric KNI that we introduce as the fraction of *k*-nearest neighbors in the embedding that are of the same type, averaged over all cells. A high KNI value indicates homogeneous neighborhoods of cell types, while a random mixing of cells would cause low KNI values. We compared the performance of JVis to conventional t-SNE and UMAP applied to the concatenation of modalities that were normalized by dividing them by the Frobenius norm of the count matrix and to the embedding obtained when assigning (fixed) uniform weights to each modality (*α*_*i*_=1/3 in (1)). Additional file 1: Figs. S7-S10 demonstrate the benefit of borrowing information across modalities by the joint optimization scheme implemented in JVis. Compared to the normalized concatenation and the (uniform) averaging approach, the distinction between meaningful and noisy modalities in j-SNE and j-UMAP yields more accurate embeddings, across various noise levels. For data sets containing 5000 cells the separation of cell types in the embeddings obtained with (fixed) uniform weights continuously decreased with increasing noise levels. In contrast, the joint optimization scheme in j-SNE was able to retain a high accuracy on these data sets (Additional file 1: Figs. S7 and S9), especially for smaller penalties assigned to non-uniform weights, i.e. small values of *λ*. This is consistent with the sharper drop in the weight associated with the noise modality observed for data sets containing 5000 cells and small values of *λ* (Additional file 1: Fig. S3). For j-UMAP, on the other hand, computed weights were close to uniform (for *λ*≥0.5) on data sets GpbmcN5k and GpbmcN5kD1k (Additional file 1: Fig. S4) and thus yielded embeddings with similar accuracy as the uniform weighting scheme on these data sets (Additional file 1: Figs. S8 and S10). The normalized concatenation approach works reasonably well on data sets with 1000 cells and large number of genes (N1k), but its performance varies substantially between different data sets and is even less accurate than its unnormalized version on data sets with 5000 cells. On most data sets, concatenation-based approaches show a sharp initial drop in accuracy for small levels of noise.

t-SNE and UMAP often produce embeddings that are in good agreement with known cell types or cell types computed by unsupervised clustering [17, 18] of high-dimensional molecular measurements such as mRNA expression. The simultaneous measurement of multiple types of molecules such as RNA and protein can refine cell types and JVis seeks to capture this refinement in their low-dimensional embedding. We compared unimodal and multimodal embeddings of mRNA and surface protein (ADT) expression measured in 4292 healthy human PBMCs [15] and in 8617 cord blood mononuclear cells (CBMC) [2] using CITE-seq [2]. Cell type labels were inferred by methods Specter [4] or CiteFuse [5], which have recently been introduced for the joint clustering of CITE-seq data.

Consistent with observations in [4, 5], t-SNE and UMAP visualizations of transcriptomic data alone does not show a clear distinction of CD4+ T cells and CD8+ T cells in the CBMC data set, while the embedding of protein expression mixes dendritic cells with CD14+ cells (Fig. 2d–f, Additional file 1: Figs. S11, S12). In contrast, JVis makes use of both modalities to compute a joint embedding that accurately separates CD4+ and CD8+ T cells as well as dendritic and CD14+ cells. Again, we confirm the visual interpretation quantitatively using the same metrics as above (Additional file 1: Table S2). The joint embedding of mRNA and ADT by JVis yields substantially larger Silhouette scores than the two unimodal t-SNE and UMAP emeddings.

Similarly, the joint embeddings of cells in the PBMC data set by JVis separate naïve and memory CD4+ T cell that are mixed in the ADT based t-SNE and UMAP embeddings as well as CD4+ and CD8+ T cells that are mixed in the mRNA based embeddings (Additional file 1: Figs. S13, S14). Again, joint embeddings are more accurate in terms of Silhouette scores than unimodal embeddings (Additional file 1: Table S2), even though overall the additional information provided by RNA measurements is limited relative to ADT counts on this data set.

RNA velocity [19] describes the rate of change of mRNA abundance estimated from the ratio of mature and pre-mRNA. While RNA velocity points to the future state of a cell, the recently introduced protein velocity [6] extends this concept and utilizes the joint measurment of RNA and protein abundance to infer the past, present, and future state of a cell. In [6], the authors used PCA and t-SNE to visualize RNA and protein velocity as well as the resulting *protein acceleration* in six PBMC data sets that were generated using four different technologies: CITE-seq, REAP-seq [20], ECCITE-seq [15] (data sets “CTCL”, a cutaneous T cell lymphoma patient, and “ctrl”, a healthy control), and 10X Genomics (data sets 1k and 10k). The authors observed strong velocity signals offered by the CITE-seq and 10x Genomics technologies, while REAP-seq and ECCITE-seq yielded noisier acceleration landscapes. Both RNA and protein velocity, however, were projected into the same t-SNE embedding of transcriptomic measurements alone, rendering their interpretation difficult. We therefore repeated the analysis of the six different data sets but projected velocities into the joint embedding of both modalities computed by JVis. The noisy acceleration landscapes observed in [6] in the ECCITE-seq and REAP-seq data sets become aligned across cell types in their joint embeddings by JVis (Fig. 2g–i and Additional file 1: Figs. S15, S16). Consistent with the improved distinction of transcriptionally similar CD4 and CD8 T cells in the joint embeddings above, acceleration landscapes in all six data sets are projected onto an embedding that more clearly separates CD4 and CD8 T cells compared to the original ones proposed in [6] (Fig. 2g–i and Additional file 1: Figs. S15-S19). RNA and protein velocities (without Bézier curve fitting for acceleration) for all six data sets are shown in Additional file 1: Figs. S20-S25.

The noisy acceleration landscapes reported in [6] for the REAP-seq and ECCITE-seq data sets might be a result of the larger number of measured surface proteins (44 and 49 antibodies versus 13 and 17 antibodies in CITE-seq and 10X, respectively) that provide a finer distinction of subpopulations of cells. In fact, we observed lower agreement between RNA and protein based clusterings for the ECCITE-seq data set shown in Fig. 2 (ARI 0.21), compared to the clusterings obtained from the two modalities in the CITE-seq data set that agree well (ARI 0.82). Since protein acceleration is computed from both RNA and protein abundances, their joint embedding can help to reduce visualization artifacts that arise when protein velocities are projected into a purely transcriptome based t-SNE embedding as in [6].

The complexity of Barnes-Hut based t-SNE is $\mathcal O (n\log n)$, where *n* is the number of input cells [21]. Although no theoretical complexity bounds have been established for UMAP, its empirical complexity is $\mathcal O(n^{1.14})$ [10]. Since in addition the alternating minimization in j-SNE and j-UMAP requires only a few iterations of (conventional) t-SNE and UMAP calculations to converge to its final estimation of modality weights (Additional file 1: Fig. S26), JVis is expected to scale well to large data sets. For example, it took JVis less than 5 minutes to compute an embedding of the 10,000 cells contained in the largest data set used in this study (10x 10k). Memory usage and running time of j-SNE and j-UMAP shown in Additional file 1: Figs. S27 and S28 as a function of number of cells with 2 and 4 simulated modalities demonstrate practicability of both approaches in the analysis of larger and more complex multimodel data sets. The scalability of our approach to large data sets can be further improved by combining it with the recently proposed FFT-accelerated Interpolation-based t-SNE method [22], that scales linearly with the number of cells.

## Conclusions

t-SNE and UMAP are routinely used to explore high-dimensional measurements of single cells in low-dimensional space. We have introduced method JVis that generalizes t-SNE and UMAP to the joint visualization of single-cell multimodal omics data. We have demonstrated that JVis combines multiple omics measurements of single cells into a unified embedding that exploits relationships among them that are not visible when applying conventional t-SNE or UMAP to each modality separately. Higher expected levels of noise in the measurements can be counteracted by smaller regularization coefficients *λ* that allow to downweight noisy modalities. Not surprisingly, projecting RNA and protein velocities into the joint embedding of both modalities yielded less noisy acceleration landscapes compared to embeddings of mRNA measurements alone. We therefore anticipate that JVis will aid in the meaningful visual interpretation of data generated by emerging multimodal omics technologies such as CITE-seq [2] and SHARE-seq [23], the latter allowing to combine RNA velocity with *chromatin potential*.

## Methods

A formal description of our generalizations j-SNE and j-UMAP as well as the algorithm to solve the underlying optimization problem can be found in Additional file 1: Supplementary Methods. The maximal number of iterations in our alternating optimization approach was set to 10 in all experiments (*maxIter*=10). Guided by the results of our simulation study and by visual inspection of known cell types, we set the regularization parameter *λ* to 3 for j-SNE and to 1 for j-UMAP in all experiments.

To simulate mRNA and ADT counts for each cell in data sets listed in Additional file 1: Table S1, we followed the strategy proposed in [13] and ran Splatter [14] with model parameters estimated from a real PBMC CITE-seq data set [15]. In particular, the same number of genes and antibodies were used, and ADT counts were simulated based on estimated dropout rate, library size, expression outlier, and dispersion across features. We added a third modality by duplicating gene expression measurements and randomly permuting expression vectors between a variable size random subset of cell. The larger the subset of cells, the larger the artificially introduced level of noise in this third modality. For the runtime and memory experiments, we generated a fourth modality by applying the same strategy to ADT counts, shuffling measurements between 40% of cells.

We measured the accuracy of an embedding using two different metrics. We introduce the k-nearest neighbor index (KNI), which denotes the fraction of *k*-nearest neighbors in the embedding that are of the same type. We used *k*=10 if not specified otherwise and computed the average across all points. Different values of *k* yielded consistent results (Additional file 1: Figs. S29-S32). In addition, we used the Silhouette score [16] that ranges between −1 and 1 to measure how much cell types overlap (score 0) or how well separated (score 1) they are. We used the Adjusted Rand Index (ARI) [24] to measure the agreement between RNA and protein based clusterings. Clusterings of cells were computed using the Louvain algorithm [17] where the resolution parameter is tuned to match the number of annotated cell types. Following best practice [25], we used standard preprocessing of the input data including log-transformation of the expression matrix followed by principal component analysis (PCA) and applied j-SNE and j-UMAP as well as their conventional counterparts to 20 or 50 principle components. In all protein velocity experiments, preprocessed data was taken from the original study [6], no further preprocessing was performed. We computed protein acceleration using the protaccel Python package introduced in [6].

## Supplementary Information

**Additional file 1** Supplementary Methods, Tables S1, S2 and Fig. S1-Fig. S32.

**Additional file 2** Review history

## Data Availability

The SNARE-seq and CBMC CITE-seq data sets were downloaded from Gene Expression Omnibus with accession codes GSE126074 and GSE126310, respectively. The six data sets used in the protein acceleration experiments were from [6]. The implementations of j-SNE and j-UMAP are based on the *scikit-learn* v0.23.1 library [26] and the UMAP v0.4.5 Python package [10], respectively. The JVis Python package can be installed through PyPi [27] and its open-source code is maintained at https://github.com/canzarlab/JVis-learn [28] under the 3-clause BSD license. The source code has been deposited in archived format at 10.5281/zenodo.4682805 [29]. Python scripts to reproduce all results in this paper are available at https://github.com/canzarlab/JVis_paper [30]. Declarations
