# Supplementary material for: A generalization of t-SNE and UMAP to single-cell multimodal omics
Source: Genome Biol. 2021 May 3;22:130. doi: 10.1186/s13059-021-02356-5 (PMC8091681; doi:10.1186/s13059-021-02356-5)
Supplement: Supplementary file 1 — Additional file 1 Supplementary Methods, Tables S1, S2 and Fig. S1-Fig. S32. [file 13059_2021_2356_MOESM1_ESM.pdf]

Supplement for: A generalization of t-SNE and UMAP to single-cell multimodal omics

Van Hoan Do[1] and Stefan Canzar*[1]

[1]Gene Center, Ludwig-Maximilians-Universität München, Munich, Germany

Contents

Supplementary Methods **2**
Generalizing t-SNE to multimodal data 2
Optimization algorithm 3
Generalizing UMAP to multimodal data 4

Supplementary Tables **6**

Supplementary Figures **7**
Proof of concept 7
JVis agrees with joint clustering 16
Protein acceleration in joint embeddings 18

*Correspondence: canzar@genzentrum.lmu.de

Supplementary Methods

Generalizing t-SNE to multimodal data

Given n data points $\mathcal{X} = \{x_1, x_2, \ldots, x_n\}$, t-SNE is a nonlinear dimensionality reduction technique that aims to learn an embedding $\mathcal{E} = \{y_1, y_2, \ldots, y_n\}$ in a low-dimensional space that preserves the distribution of point similarities. Based on a given metric d that measures the dissimilarity between pairs of points, t-SNE first computes joint probabilities p_{ij} that quantify the similarity between x_i and x_j:

$$p_{j|i} = \frac{\exp(-d(x_i, x_j)^2/2\sigma_i^2)}{\sum_{k \neq i} \exp(-d(x_i, x_k)^2/2\sigma_i^2)}, \qquad p_{i|i} = 0, \tag{1}$$

$$p_{ij} = \frac{p_{j|i} + p_{i|j}}{2n}, \tag{2}$$

where σ_i is the bandwidth of the Gaussion kernels centered at data point x_i. Given a predefined *perplexity* u, bandwidths σ_i are chosen such that the perplexity of conditional distributions P_i equal to u. The perplexity is often set to a value in the range between 5 and 50 [1]. t-SNE uses the normalized Student-t kernel with a single degree of freedom to measure similarities q_{ij} in the embedding $\mathcal{E}$:

$$q_{ij} = \frac{(1 + \|y_i - y_j\|^2)^{-1}}{\sum_{k \neq i}(1 + \|y_i - y_k\|^2)^{-1}}, \qquad q_{ii} = 0. \tag{3}$$

To determine the location of points in the embedding $\mathcal{E}$ that preserve the original similarities p_{ij} as well as possible, t-SNE seeks to minimize the Kullback-Leibler (KL) divergence of distribution $P = (p_{ij})$ from distribution $Q = (q_{ij})$.

$$C(\mathcal{E}) = KL(P||Q) = \sum_{i \neq j} p_{ij} \log \frac{p_{ij}}{q_{ij}}. \tag{4}$$

The minimization of equation (4) is performed using a gradient descent algorithm. The gradient is given by

$$\frac{\partial C}{\partial y_i} = 4 \sum_{j \neq i} (p_{ij} - q_{ij}) q_{ij} Z (y_i - y_j), \tag{5}$$

where $Z = \sum_{k \neq l}(1 + \|y_k - y_l\|^2)^{-1}$. While a naive implementation of t-SNE has runtime complexity $\mathcal{O}(n^2)$, the algorithm proposed in [2] uses the Barnes-Hut algorithm [3] and a sparse approximation of point similarities to reduce its complexity to $\mathcal{O}(n \log n)$ which allows it to scale to data sets comprising hundreds of thousands of cells.

We generalize t-SNE to the joint dimensionality reduction of multimodal data as follows. Given K-modal data points $X = \{x_1^{(k)}, x_2^{(k)}, \ldots, x_n^{(k)}\}, k = 1, 2, \ldots, K$, we want to learn a joint embedding $\mathcal{E} = \{y_1, y_2, \ldots, y_n\}$ that preserves similarities between points in X, analog

to t-SNE, but in all modalities at the same time. Here, joint probability p_{ij}^k measures the similarity of points x_i^k and x_j^k with respect to modality k. Generalizing the objective of t-SNE, we aim to minimize the KL divergence of distributions $P^{(k)}$ for each modality k from distribution Q:

$$C(\mathcal{E}) = \sum_k \alpha_k KL(P^{(k)}||Q) + \lambda \sum_k \alpha_k \log \alpha_k \tag{6}$$

$$= \sum_k \sum_{i \neq j} \alpha_k p_{ij}^{(k)} \log \frac{p_{ij}^{(k)}}{q_{ij}} + \lambda \sum_k \alpha_k \log \alpha_k, \tag{7}$$

where $\alpha_k \geq 0$, $\sum_k \alpha_k = 1$, and $\lambda > 0$ is a regularization parameter. The first term of (6) denotes a convex combination of $\mathrm{KL}(P^{(k)}||Q), k = 1, 2, \ldots, K$, in which coefficients α_k represents the importance (contribution) of modality k to the joint embedding. That is, the larger α_k, the stronger the influence of modality k on the final location of points in the embedding. The second term of the objective function is a regularization term that prevents the joint embedding from being biased towards individual modalities.

Optimization algorithm

We use an alternating optimization scheme to minimize (7) jointly over the location of points y_i and the weighting of modalities α. In each iteration, we first fix α and use t-SNE to find points y in the joint embedding, after which we find the best (according to (7)) weighting α for fixed points y.

Initialization of α. We initially give uniform weights α to all modalities, i.e.,

$$\alpha_k = 1/K, \text{ for all } k = 1, 2, \ldots, K. \tag{8}$$

Step 1: Fix α and optimize over y. Similar to conventional t-SNE, we employ the gradient descent algorithm to minimize (7). The gradient is given by:

$$\frac{\partial C}{\partial y_i} = 4 \sum_k \sum_{j \neq i} \alpha_k (p_{ij}^{(k)} - q_{ij}) q_{ij} Z (y_i - y_j) = 4 \sum_{j \neq i} \big(\sum_k \alpha_k p_{ij}^{(k)} - q_{ij} \big) q_{ij} Z (y_i - y_j). \tag{9}$$

Comparing gradient (9) to the original gradient (5), the former can be obtained by replacing distribution P used in (5) by the convex combination of distributions $P^{(k)}$, i.e., $P = \sum_k \alpha_k P^{(k)}$. In other words, the joint t-SNE of multiple modalities can be computed by applying conventional (unimodal) t-SNE to the convex combination of distributions $P^{(k)}$ for all modalities k.

Step 2: Fix y and optimize over α. Given a joint embedding of points y, problem (7) is a special case of the following problem:

$$\begin{aligned} \min_{w} \quad & b_i\alpha_i + \lambda \sum_k \alpha_k \log \alpha_k \\ \text{subject to} \quad & \sum_k \alpha_k = 1, \\ & \alpha_k \geq 0, \qquad k = 1, 2, \ldots, K. \end{aligned} \tag{10}$$

This problem has a convex objective and linear constraints. We derive a closed form solution using the Karush-Kuhn-Tucker (KKT) conditions. The Lagrangian function corresponding to the constrained optimization problem (10) is given by

$$\mathcal{L}(\alpha, u, v) = b_i\alpha_i + \lambda \sum_k \alpha_k \log \alpha_k + u\big(\sum_k \alpha_k - 1\big) - \sum_k v_k\alpha_k.$$

The KKT conditions are given by

$$\begin{cases} \frac{\partial \mathcal{L}(\alpha,u,v)}{\partial \alpha_k} = b_k + \lambda(1 + \log \alpha_k) + u - v_k = 0, \\ v_k\alpha_k = 0, \\ \sum_k \alpha_k = 1, \alpha_k \geq 0, \\ v_k \geq 0, \end{cases}$$

for all $k = 1, 2, \ldots, K$. Assuming non-zero contributions of each modality, i.e. strictly positive values for α, we have $v_k = 0$ for all k and $\alpha_k = \exp\{(-b_i - u)/\lambda - 1\}$. Together with the constraint $\sum_k \alpha_k = 1$, we obtain

$$\alpha_k = \frac{m_k}{\sum_k m_k},$$

where $m_k = \exp\{-b_i/\lambda - 1\}$. We alternate iteratively between steps 1 and 2 until the improvement in the objective value falls below a predefined error threshold ϵ or until a maximum number *maxIter* of iterations has been reached. Note that distributions $P^{(k)}$ do not need to be recompute after the first iteration, speeding up later iterations of j-SNE. Furthermore, when $\lambda = 0$, the optimal value of problem (10) is $\min_i\{b_i\}$, and an optimal solution is $\alpha_{i^*} = 1, \alpha_j = 0$ for all $j \neq i^*$, where $b_{i^*} = \min_i\{b_i\}$. This solution is unique if $b_{i^*} = \min_i\{b_i\}$ is unique. In this case, the optimal joint embedding will converge to the optimal embedding of a single modality.

Generalizing UMAP to multimodal data

UMAP uses different definitions of high and low-dimensional similarities p_{ij} and q_{ij}, respectively. In particular, UMAP similarities $p_{j|i}$ are defined and symmetrized to give p_{ij} as follows:

$$p_{j|i} = \exp[(-d(x_i, x_j) - \rho_i)/\sigma_i)], \qquad p_{i|i} = 0,$$

$$p_{ij} = p_{j|i} + p_{i|j} - p_{j|i}p_{i|j},$$

where ρ_i is the distance to the nearest neighbor of x_i, and σ_i is the normalizing factor which is found through binary search using a criteria similar to the perplexity-based selection of bandwidts in t-SNE. The low-dimensional similarities are defined as:

$$q_{ij} = \left(1 + a \left\|y_i - y_j\right\|_2^{2b}\right)^{-1},$$

where a and b are user-defined parameters with default values $a \approx 1.929$ and $b \approx 0.7915$. Note that setting $a = b = 1$ gives the Student t-distribution used to define low-dimensional similarities in t-SNE (equation (3)). In contrast to t-SNE, however, p_{ij} and q_{ij} are not further normalized. UMAP determines the low dimensional embedding by minimizing the cross entropy:

$$C(\mathcal{E}) = \mathrm{CE}(P, Q) = \sum_{i \neq j} p_{ij} \log\left(\frac{p_{ij}}{q_{ij}}\right) + (1 - p_{ij}) \log\left(\frac{1 - p_{ij}}{1 - q_{ij}}\right), \tag{11}$$

which is equivalent to the following objective function (ignoring constant terms):

$$C(\mathcal{E}) = \sum_{i \neq j} -p_{ij} \log q_{ij} - (1 - p_{ij}) \log(1 - q_{ij}). \tag{12}$$

Objective (12) can be minimized by stochastic gradient descent. Note, however, that UMAP uses negative sampling when trying to optimize (12) which can have a large effect on the final embedding [4].

The generalization of UMAP to multimodal data is analogous to that of t-SNE. In particular, we minimize the cross entropy between distributions $P^{(k)}$ for each modality k and distribution Q:

$$C(\mathcal{E}) = \sum_k \alpha_k \mathrm{CE}(P^{(k)} || Q) + \lambda \sum_k \alpha_k \log \alpha_k, \tag{13}$$

where, as in the joint t-SNE objective (6), the first term denotes a convex combination with coefficients α_i and the second regularization term is weighted with parameter $\lambda > 0$.

Similar to j-SNE, we use an alternating optimization approach to minimize objective (13). In contrast to t-SNE, UMAP does not normalize distributions P and Q (compare equations (1)-(3)). To be able to set λ to an identical value across experiments, we therefore normalize coefficients in the first term of (13) by their maximum value computed in the first iteration. Specifically, we optimize function $\frac{10}{c} \sum_k \alpha_k \mathrm{CE}(P^{(k)} || Q) + \lambda \sum_k \alpha_k \log \alpha_k$, where $c = \max_k \{\mathrm{CE}(P^{(k)} || Q)\}$ is a constant computed in the first iteration.

Supplementary Tables

Table S1: Overview of the simulated data sets used in this study. Data sets were simulated using Splatter and vary in number of cells (#Cells), number of genes (#Genes), number of antibodies (#Ab), number of clusters (k), and the relative abundance of cell types that were either equal, or based on cell type abundances among peripheral blood mononuclear cells (PBMCs) in healthy individuals.

Name	#Cells (N)	#Genes (D)	#Ab	k	Relative abundances (G)
GeqN1k	1,000	33,538	49	5	(0.2, 0.2, 0.2, 0.2, 0.2)
GeqN5k	5,000	33,538	49	5	
GeqN1kD1k	1,000	1,000	49	5	
GeqN5kD1k	5,000	1,000	49	5	
GpbmcN1k	1,000	33,538	49	5	PBMCs: DC: 0.02, NK: 0.2, B: 0.1 Mono: 0.08, T: 0.6
GpbmcN5k	5,000	33,538	49	5	
GpbmcN1kD1k	1,000	1,000	49	5	
GpbmcN5kD1k	5,000	1,000	49	5	

Table S2: Comparison of joint and unimodal embeddings on the PBMC and CBMC data sets. KNI denotes the fraction on k-nearest neighbors in the embedding that are of the same type, averaged over all cells. Larger Silhouette scores indicate a better separation of cell types. Only cells assigned identical labels based on joint clusterings by CiteFuse and Specter are considered in the evaluation.

	CBMC		PBMC	
Method	KNI	Silhouette	KNI	Silhouette
j-SNE	**0.998**	**0.432**	**0.985**	**0.383**
RNA based t-SNE	0.978	0.343	0.960	0.196
ADT based t-SNE	0.989	0.270	0.949	0.366
j-UMAP	**0.998**	**0.578**	**0.985**	**0.525**
RNA based UMAP	0.980	0.487	0.960	0.111
ADT based UMAP	0.982	0.468	0.948	0.511

Supplementary Figures

Proof of concept

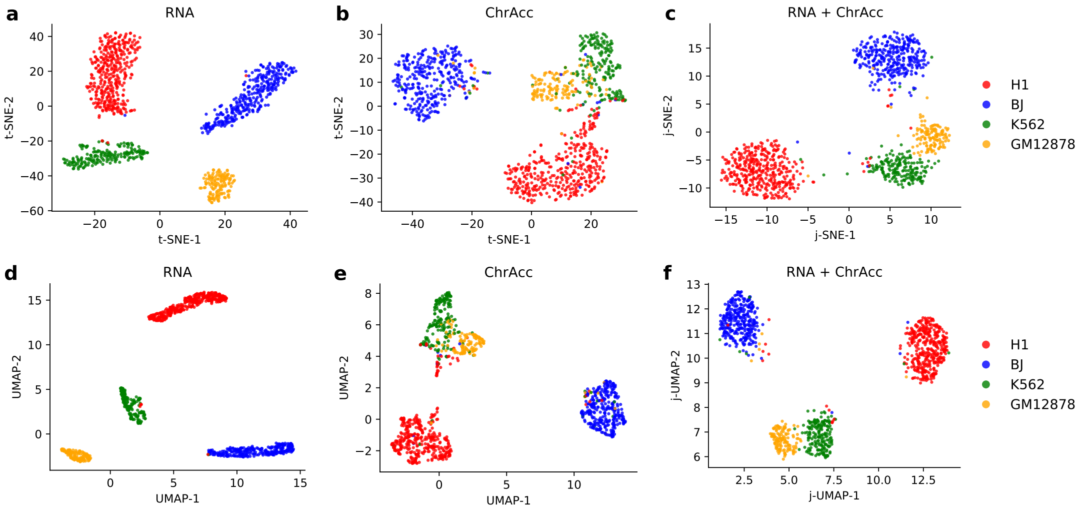

Fig. S1: Unimodal and multimodal visualization of SNARE-seq measurements. Accessible chromatin (ChrAcc) and gene expression was measured simultaneously in single cell from human cell lines BJ, H1, K562, and GM12878. *First row:* Comparison of t-SNE and j-SNE. Conventional t-SNE embedding of measurements of (**a**) RNA or (**b**) accessible chromatin. (**c**) Joint embedding of both modalities (RNA and chromatin accessibility) by j-SNE. *Second row:* Comparison of UMAP and j-UMAP. Conventional UMAP of (**d**) RNA or (**e**) accessible chromatin. (**f**) Joint embedding of both modalities by j-UMAP.

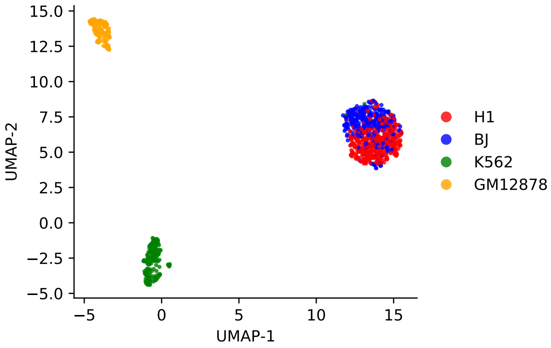

Fig. S2: Conventional UMAP embedding of cells based on SNARE-seq gene expression measurements that were randomly shuffled between human cell lines BJ and H1.

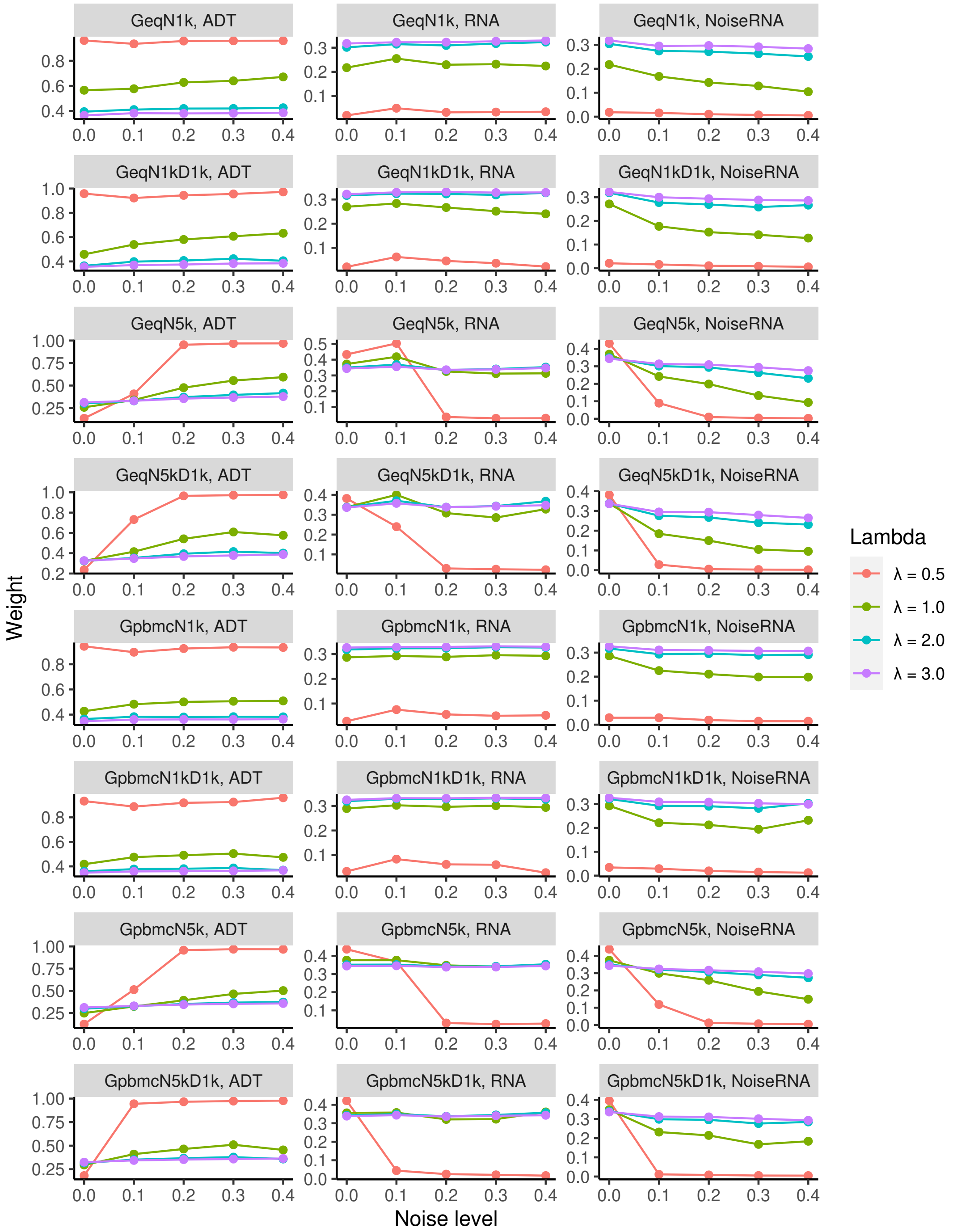

Fig. S3: j-SNE modality weights for eight simulated data sets as function of noise. Weights (α) for modalities ADT (left), RNA (middle), and NoiseRNA (right) were computed using different regularization coefficients λ. Each data set is shown in one row.

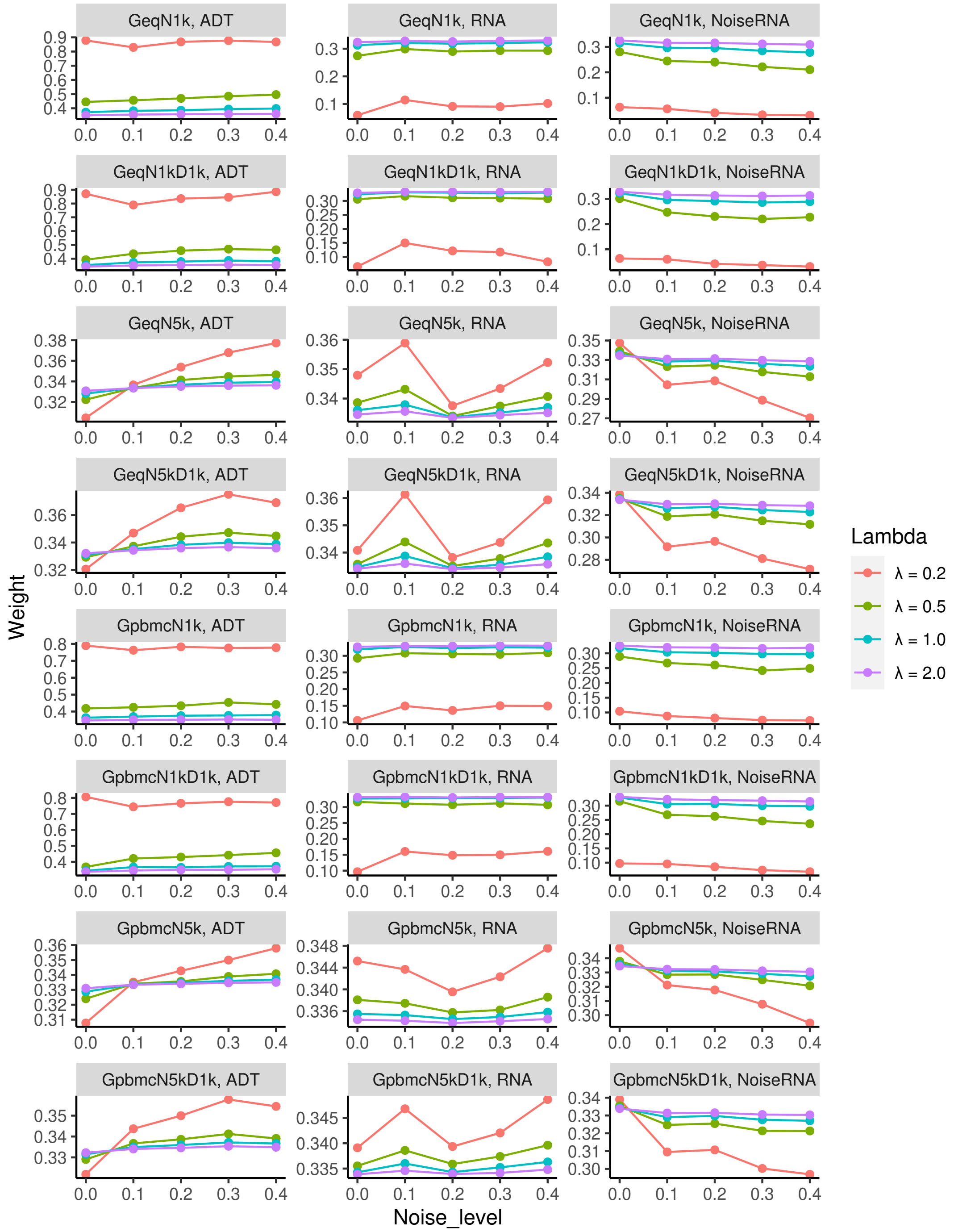

Fig. S4: j-UMAP modality weights for eight simulated data sets as function of noise. Weights (α) for modalities ADT (left), RNA (middle), and NoiseRNA (right) were computed using different regularization coefficients λ. Each data set is shown in one row.

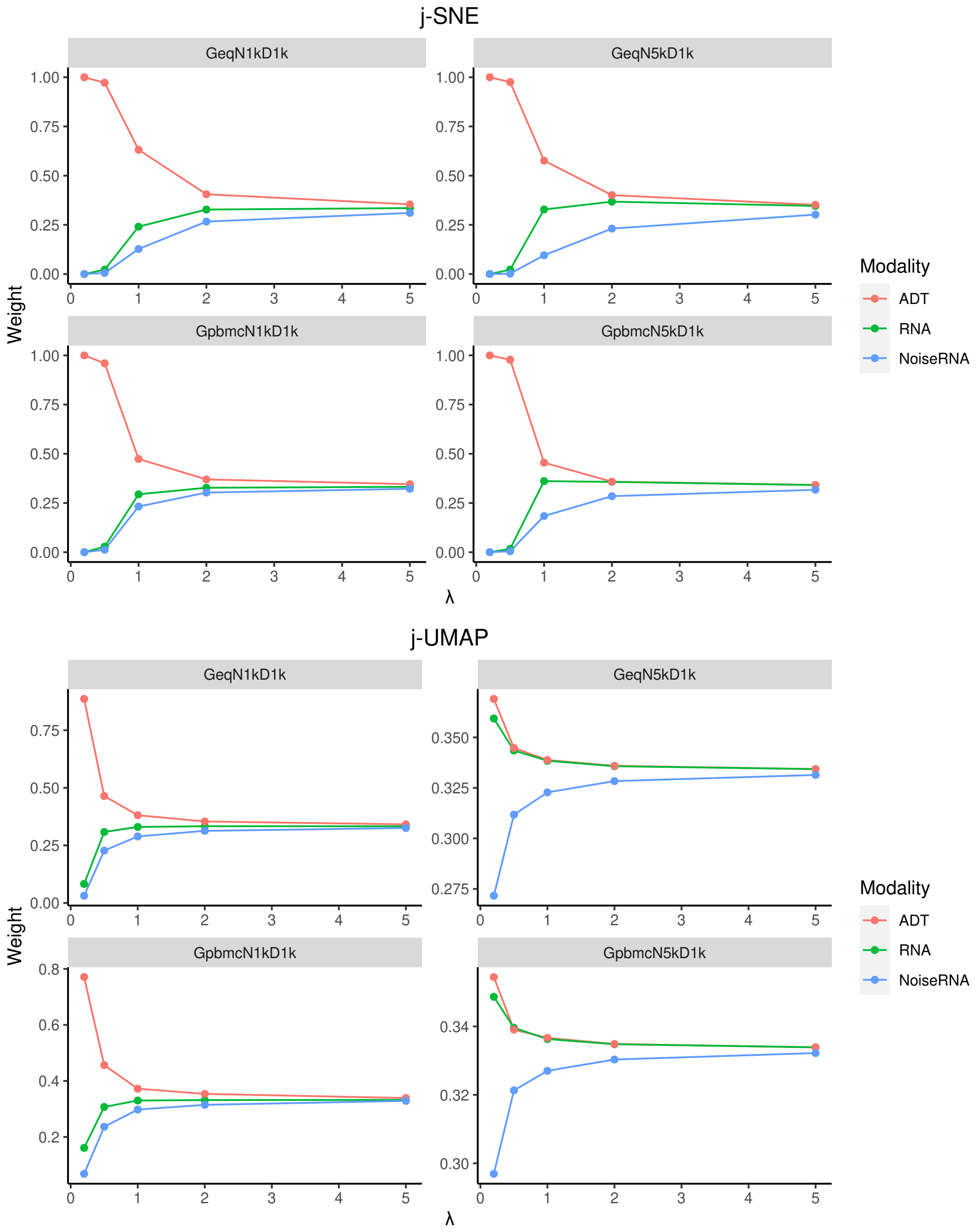

Fig. S5: Weights of modalities ADT, RNA, and NoiseRNA computed in j-SNE (top) and j-UMAP (bottom) as a function of regularization coefficient λ ranging from $0.2 - 5$. The noise level in the four simulated data sets is held constant at 0.4.

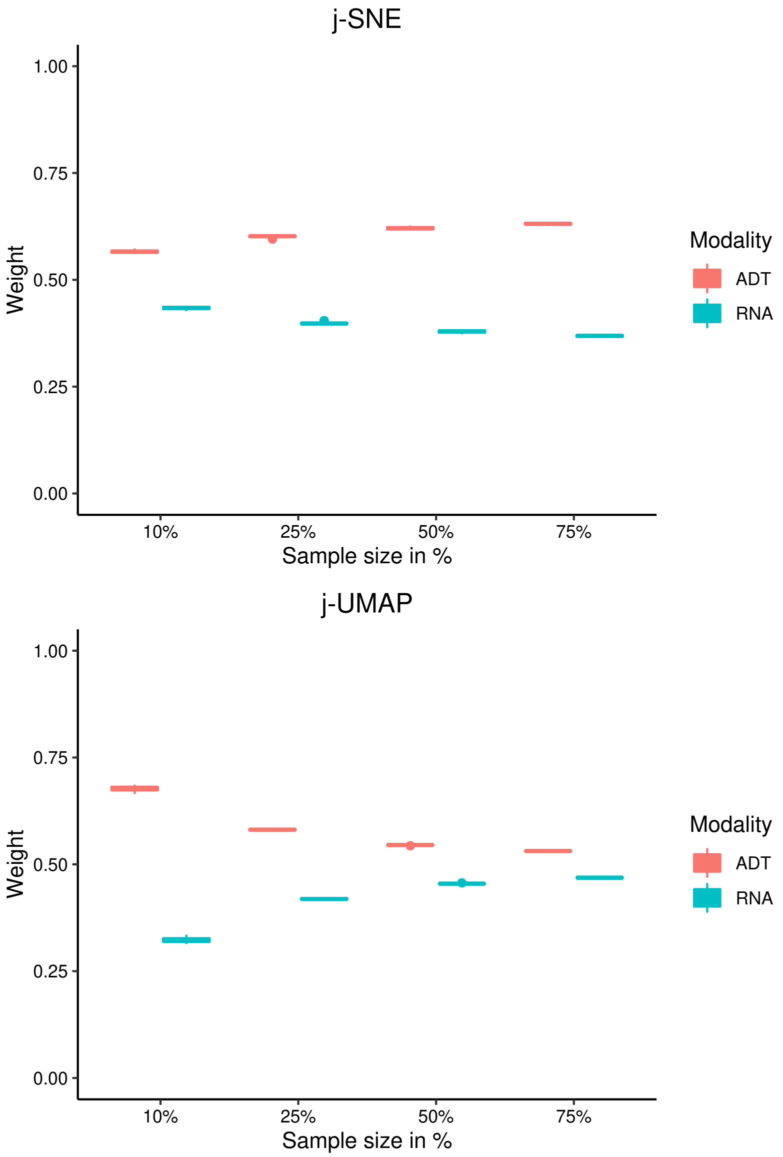

Fig. S6: Stability of computed weights. Weights of modalities ADT and RNA were computed by j-SNE (top) and j-UMAP (bottom) in 10 random trials on subsample sizes ranging from 10% to 75% of cells in simulated data set GeqN5kD1k.

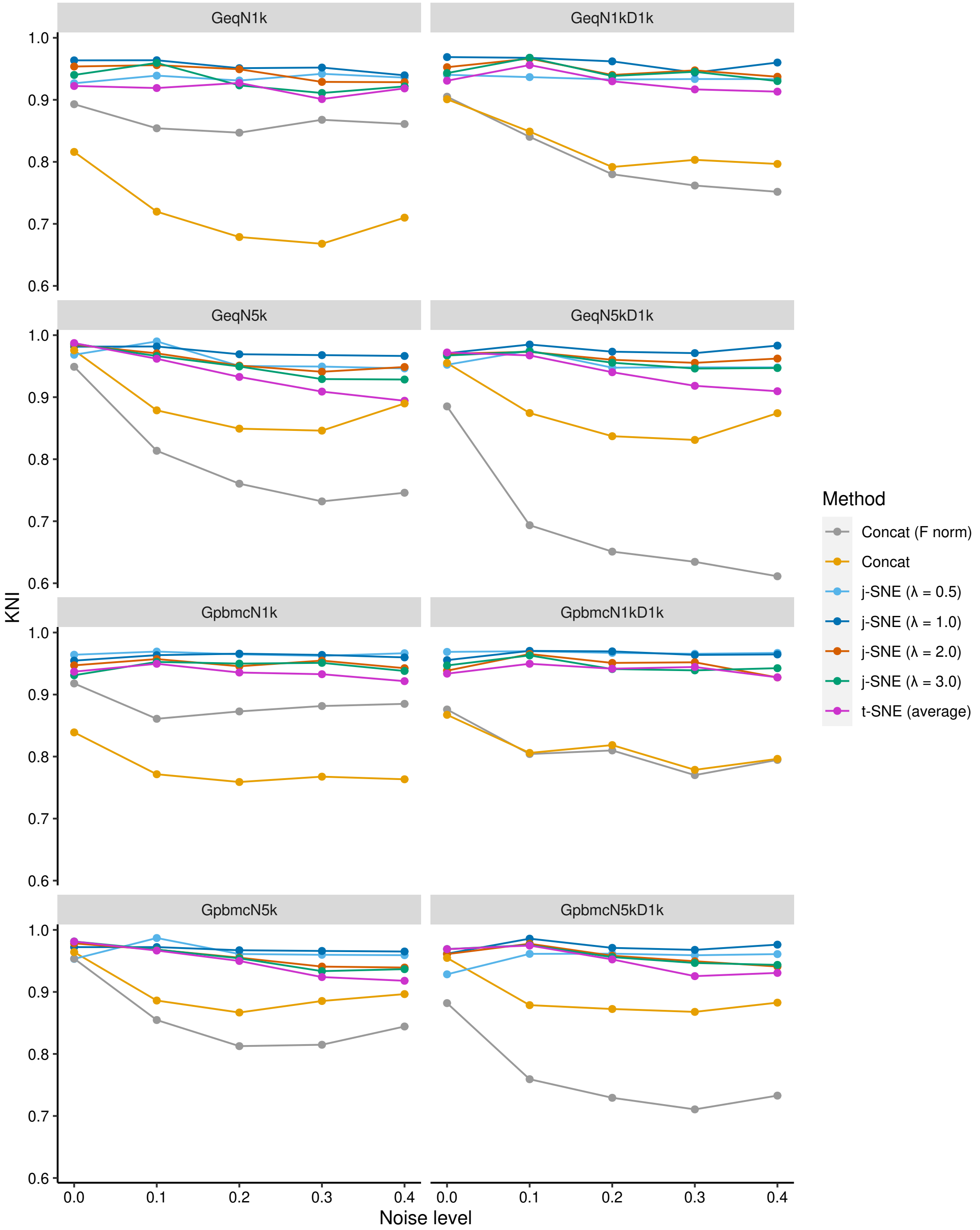

Fig. S7: KNI values of embeddings computed by j-SNE and alternative methods on eight simulated data sets. Values are shown as a function of noise for different regularization coefficient λ used in j-SNE. Conventional t-SNE is run for uniform weights assigned to each modality ($\alpha_i = 1/3$) (t-SNE (average)), or on concatenated modalities (Concat) that are optionally normalized by the Frobenius norm (Concat (F norm)).

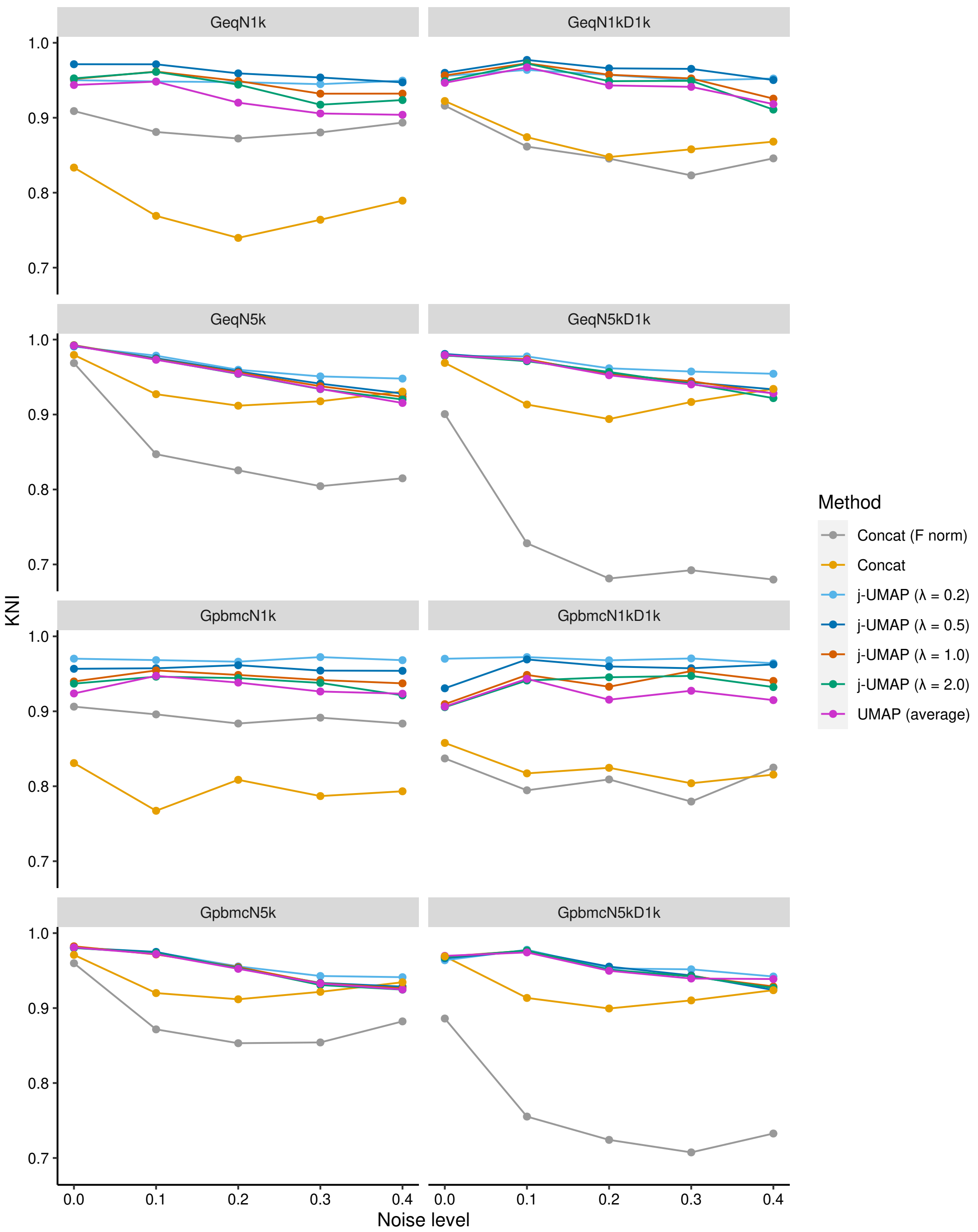

Fig. S8: KNI values of embeddings computed by j-UMAP and alternative methods on eight simulated data sets. Values are shown as a function of noise for different regularization coefficient λ used in j-UMAP. Conventional UMAP is run for uniform weights assigned to each modality ($\alpha_i = 1/3$) (UMAP (average)), or on concatenated modalities (Concat) that are optionally normalized by the Frobenius norm (Concat (F norm)).

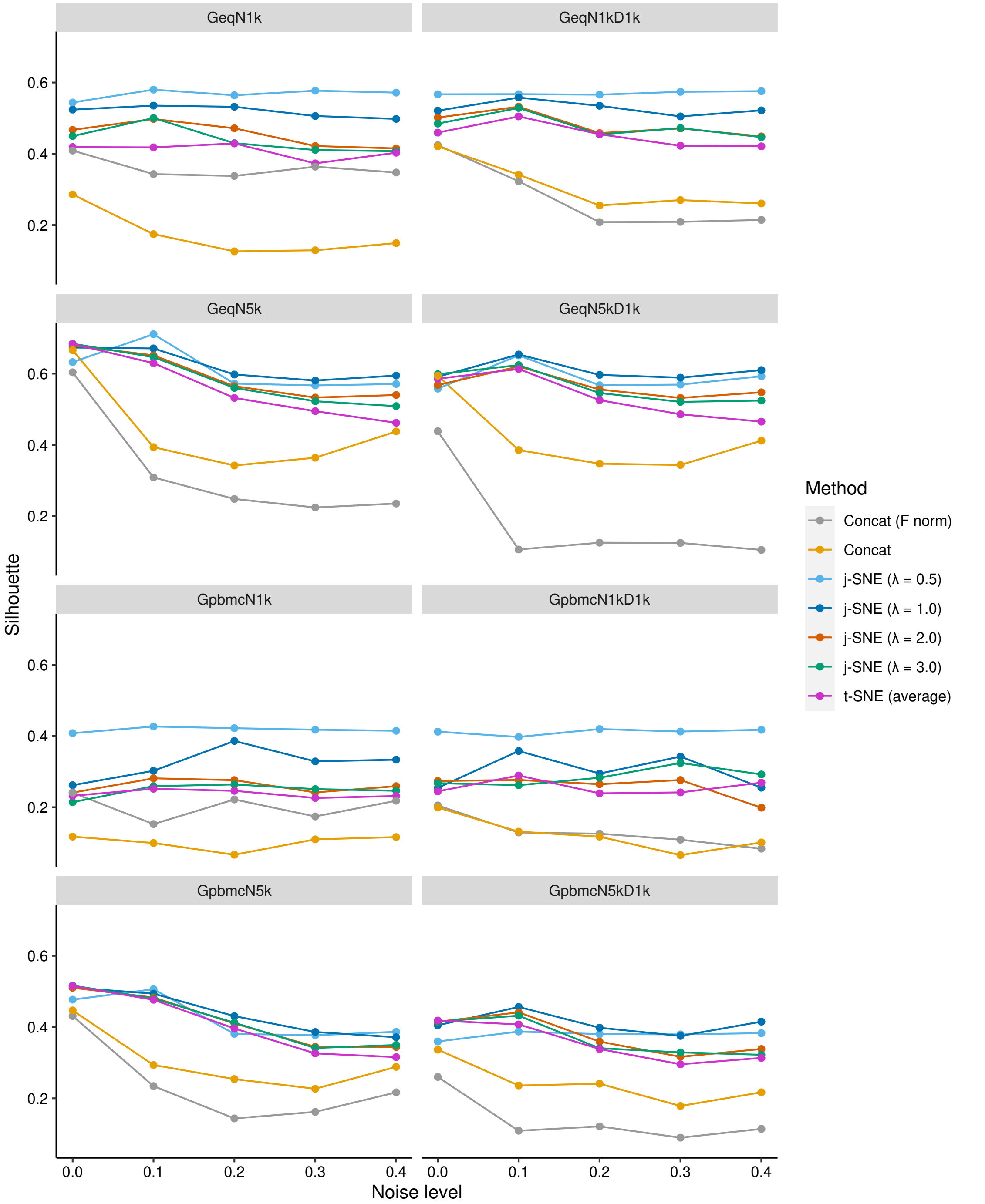

Fig. S9: Silhouette scores of embeddings computed by j-SNE and alternative methods on eight simulated data sets. Values are shown as a function of noise for different regularization coefficient λ used in j-SNE. Conventional t-SNE is run for uniform weights assigned to each modality ($\alpha_i = 1/3$) (t-SNE (average)), or on concatenated modalities (Concat) that are optionally normalized by the Frobenius norm (Concat (F norm)).

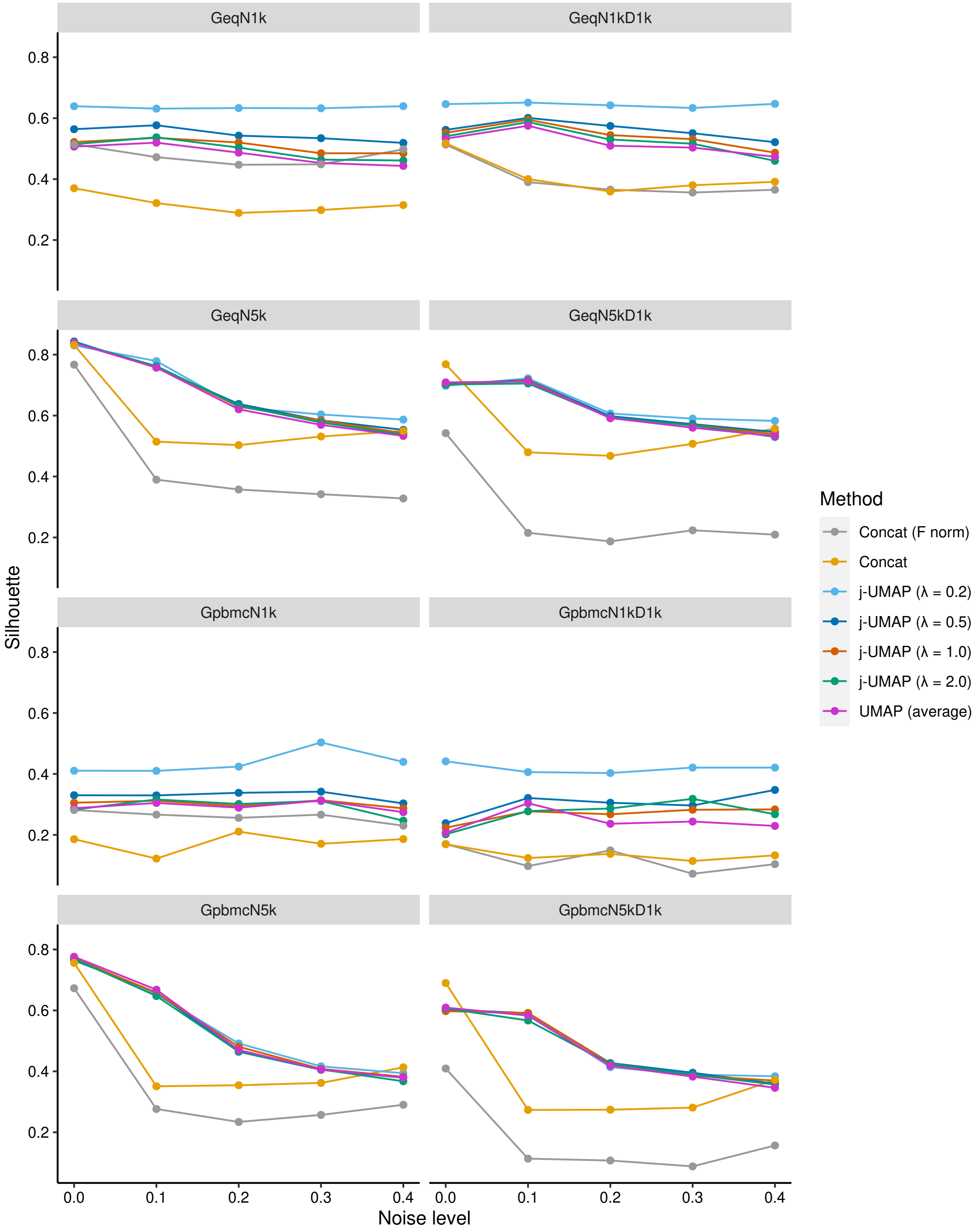

Fig. S10: Silhouette scores of embeddings computed by j-UMAP and alternative methods on eight simulated data sets. Values are shown as a function of noise for different regularization coefficient λ used in j-UMAP. Conventional UMAP is run for uniform weights assigned to each modality ($\alpha_i = 1/3$) (UMAP (average)), or on concatenated modalities (Concat) that are optionally normalized by the Frobenius norm (Concat (F norm)).

JVis agrees with joint clustering

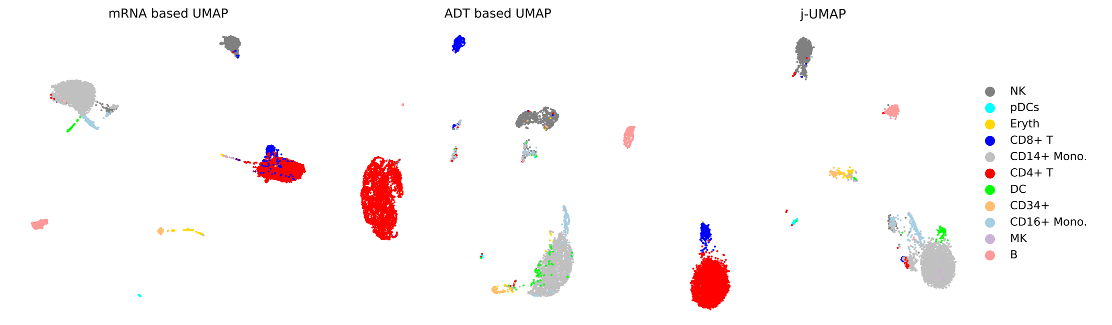

Fig. S11: UMAP/j-UMAP visualization of CBM cells. Cluster labels were identified by Specter. Embeddings were computed from mRNA measurements alone (left), protein expression (ADT) alone (middle), or jointly from both (right).

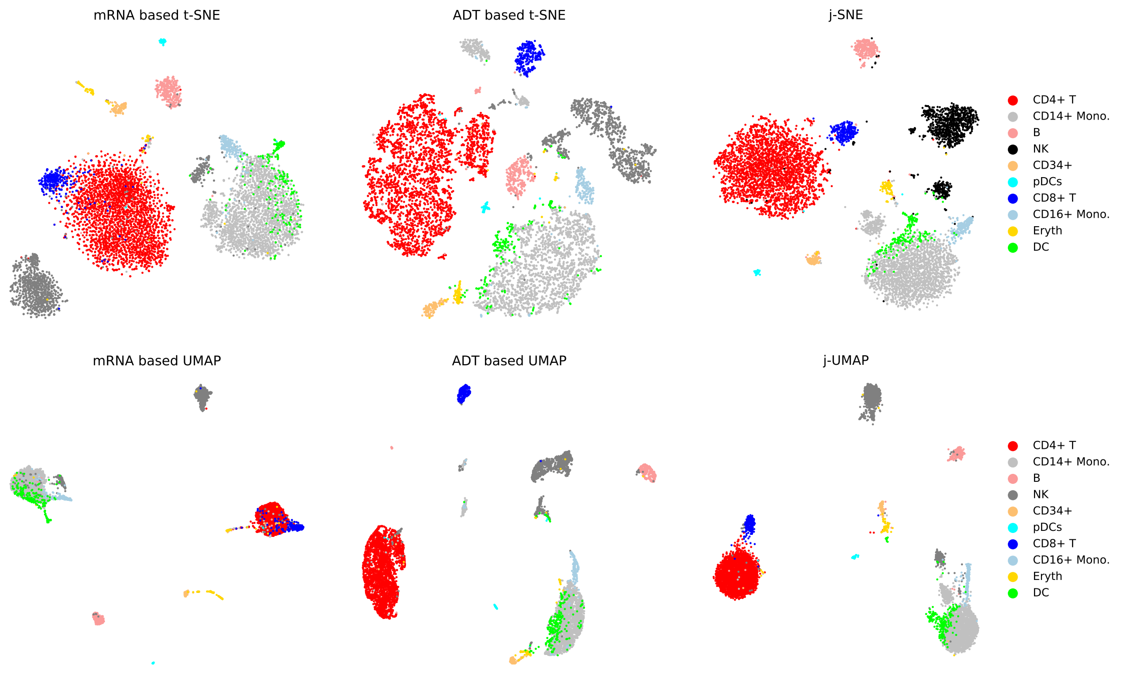

Fig. S12: t-SNE/j-SNE (top row) and UMAP/j-UMAP (bottom row) visualizations of CBM cells. Cluster labels were identified by CiteFuse. Embeddings were computed from mRNA measurements alone (left), protein expression (ADT) alone (middle), or jointly from both (right).

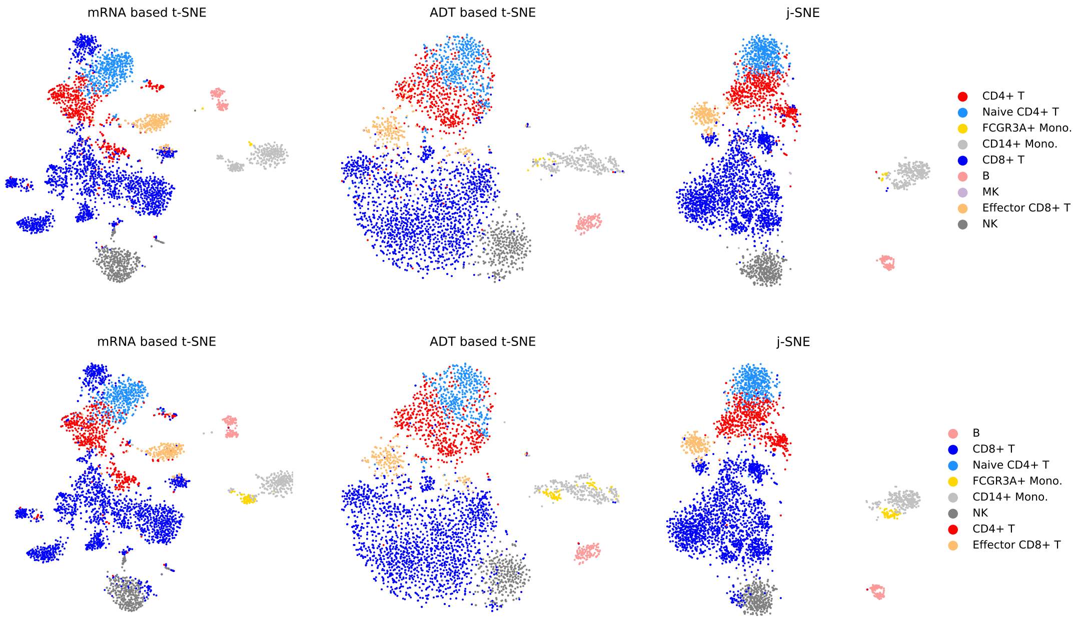

Fig. S13: t-SNE/j-SNE visualizations of PBM cells. Cluster labels were identified by Specter (top row) or CiteFuse (bottom row). Embeddings were computed from RNA measurements alone (left), protein expression (ADT) alone (middle), or jointly from both (right).

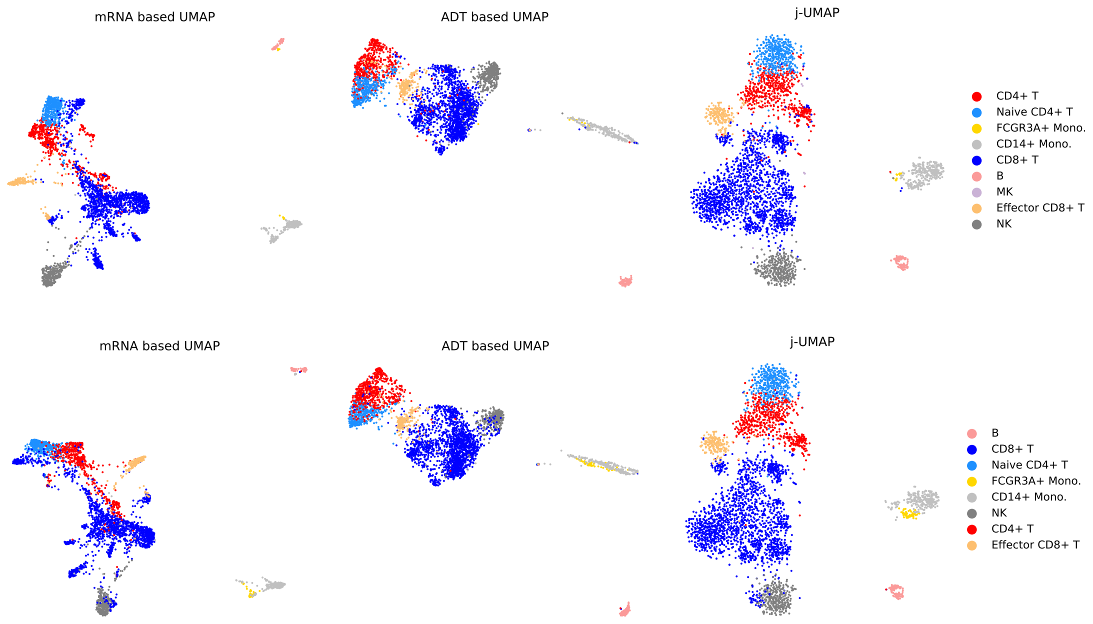

Fig. S14: UMAP/j-UMAP visualization of PBM cells. Cluster labels were identified by Specter (top row) or CiteFuse (bottom row). Embeddings were computed from RNA measurements alone (left), protein expression (ADT) alone (middle), or jointly from both (right).

Protein acceleration in joint embeddings

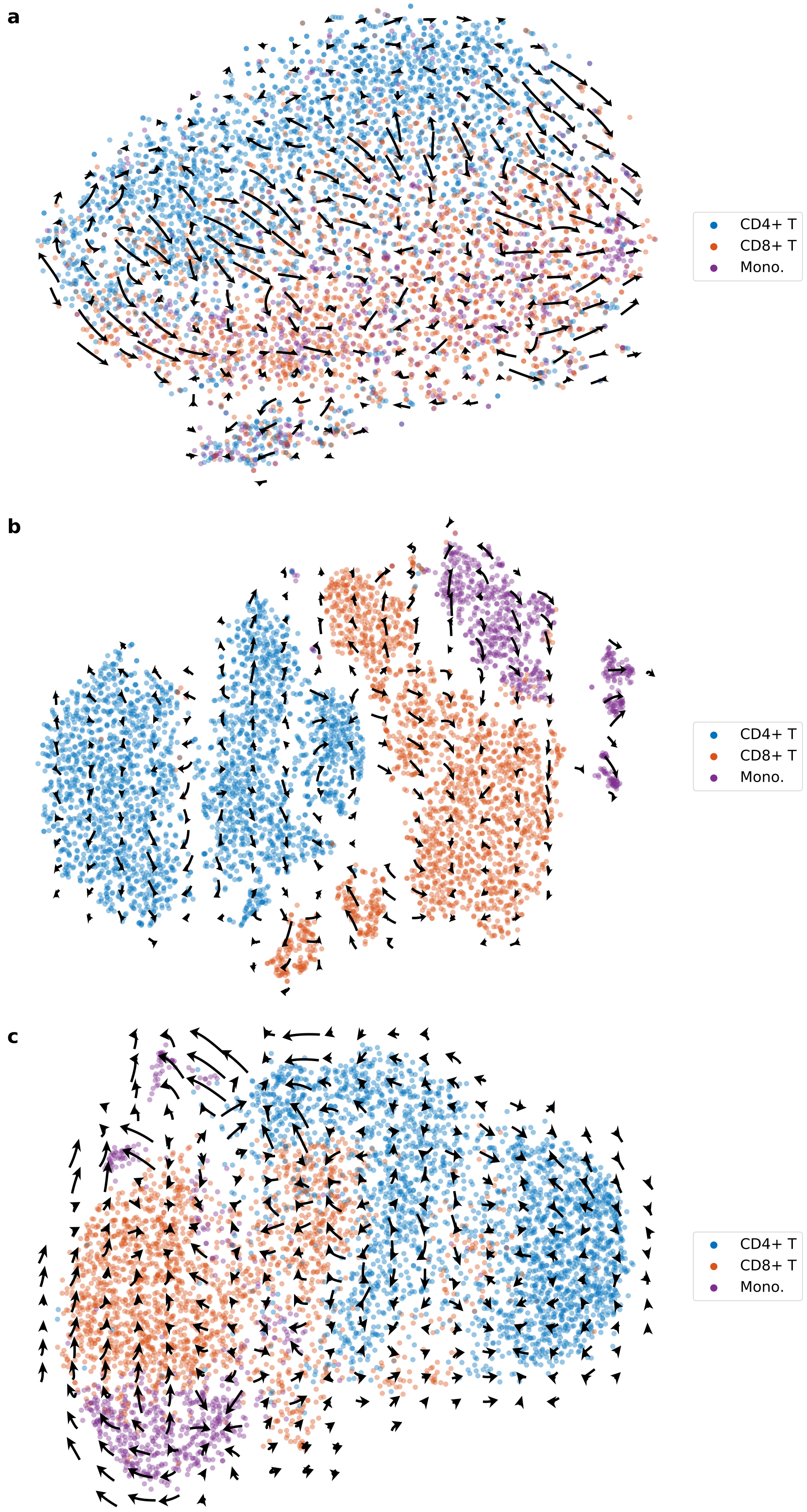

Fig. S15: Protein acceleration in ECCITE-seq data set CTCL projected into transcriptome-based t-SNE (a), j-SNE (b), and j-UMAP (c) embeddings.

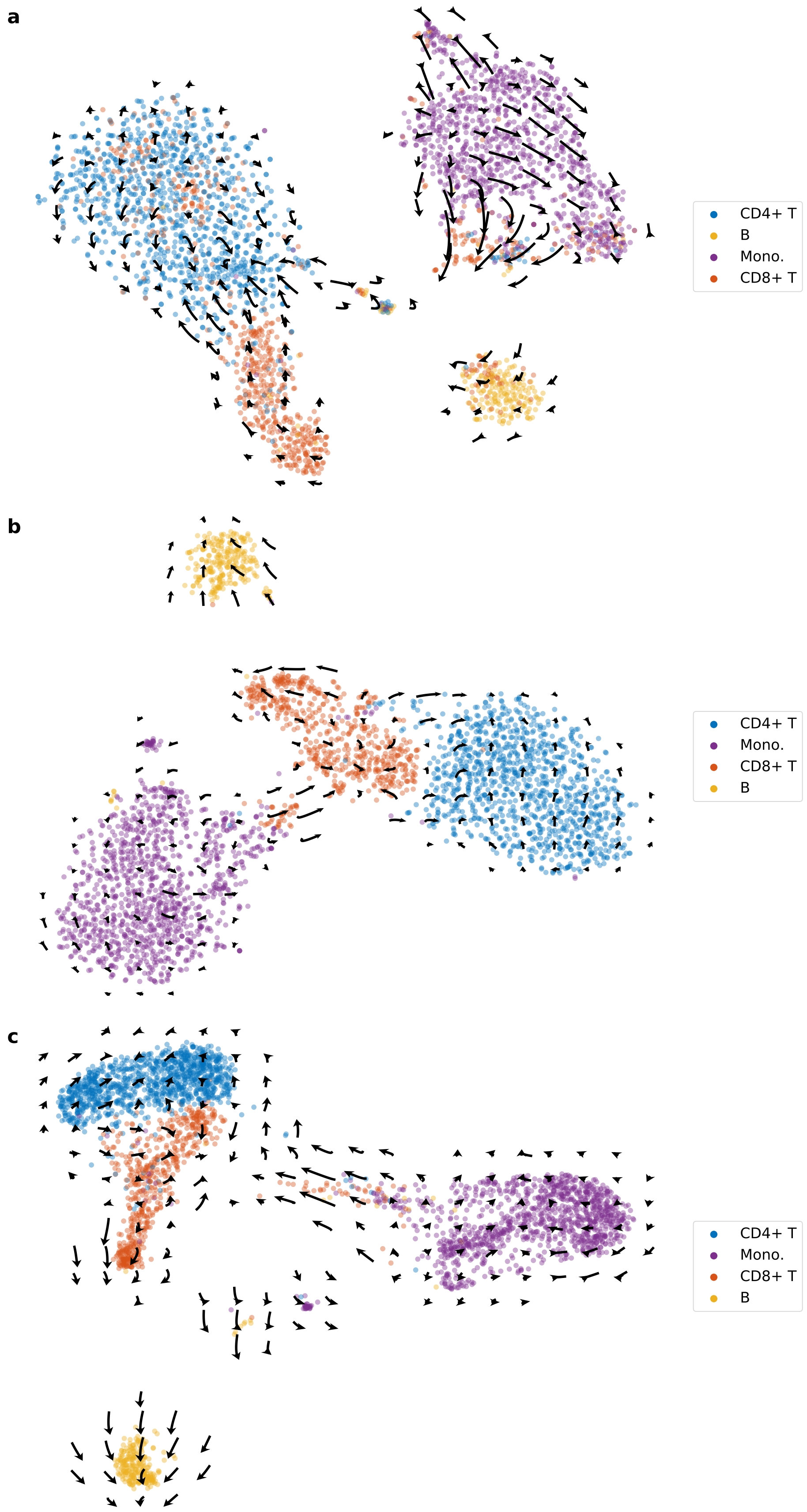

Fig. S16: Protein acceleration in REAP-seq data set projected into transcriptome-based t-SNE (a), j-SNE (b), and j-UMAP (c) embeddings.

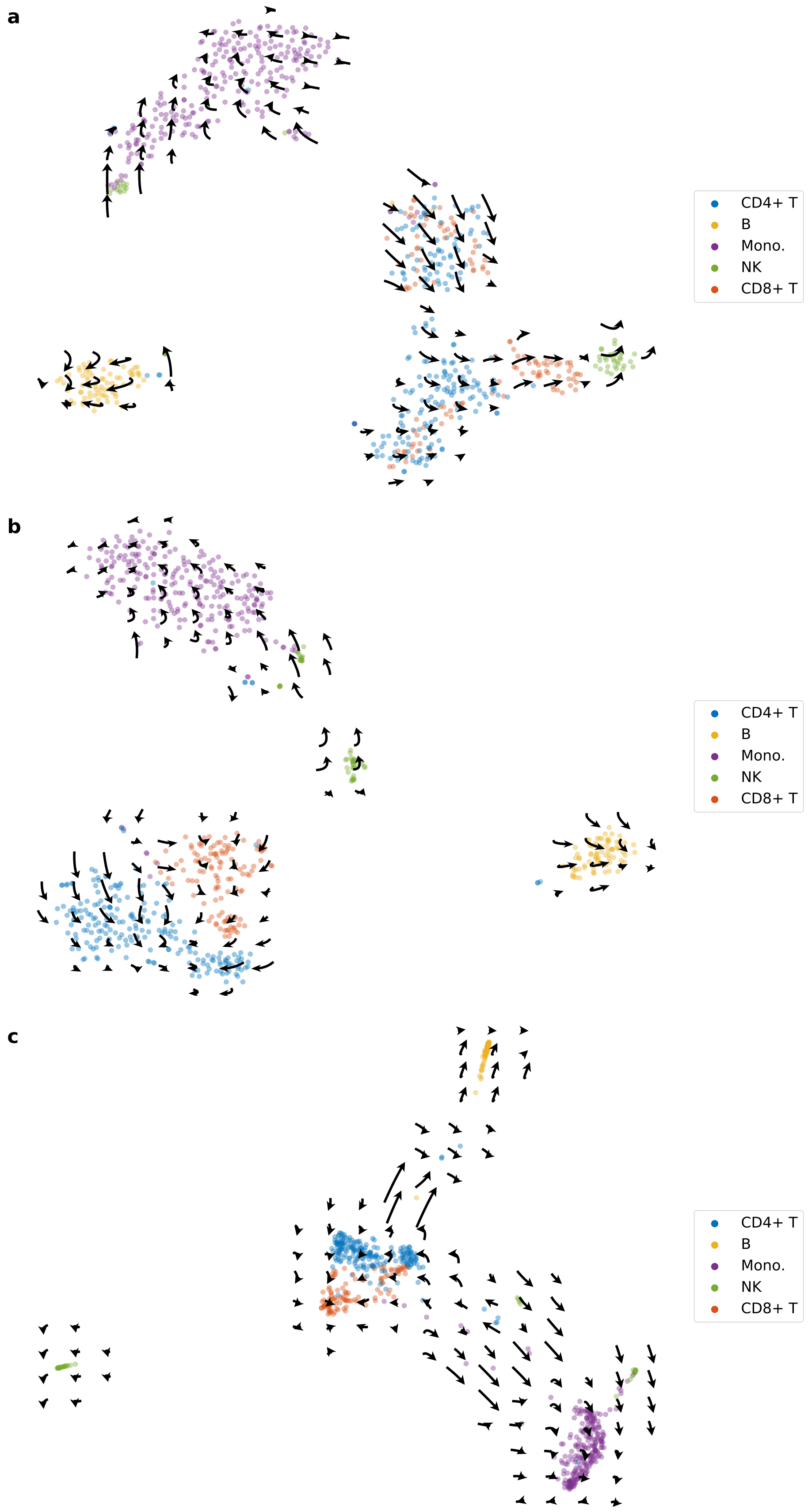

Fig. S17: Protein acceleration in 10X 1k data set projected into transcriptome-based t-SNE (a), j-SNE (b), and j-UMAP (c) embeddings.

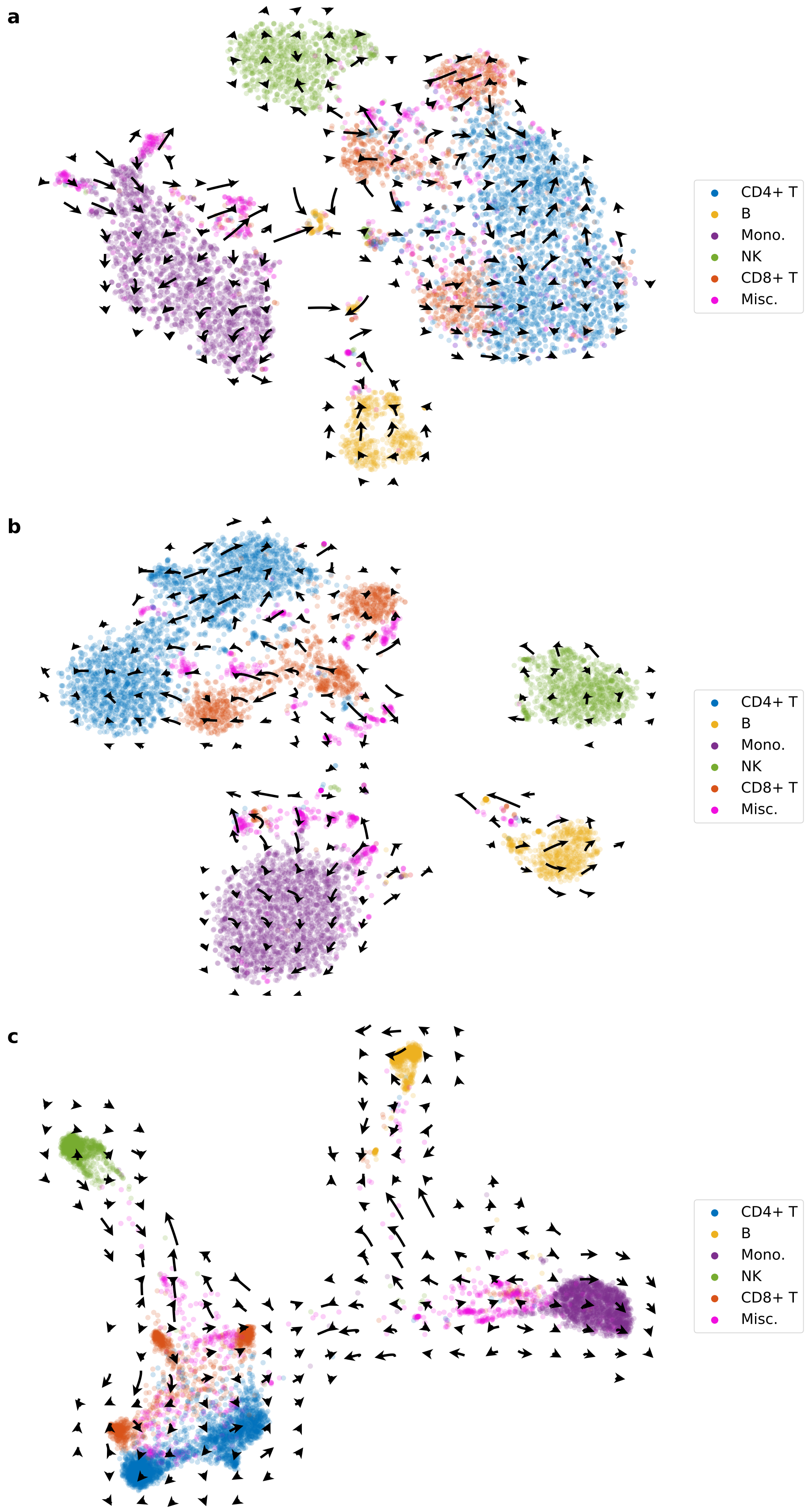

Fig. S18: Protein acceleration in 10X 10k data set projected into transcriptome-based t-SNE (a), j-SNE (b), and j-UMAP (c) embeddings.

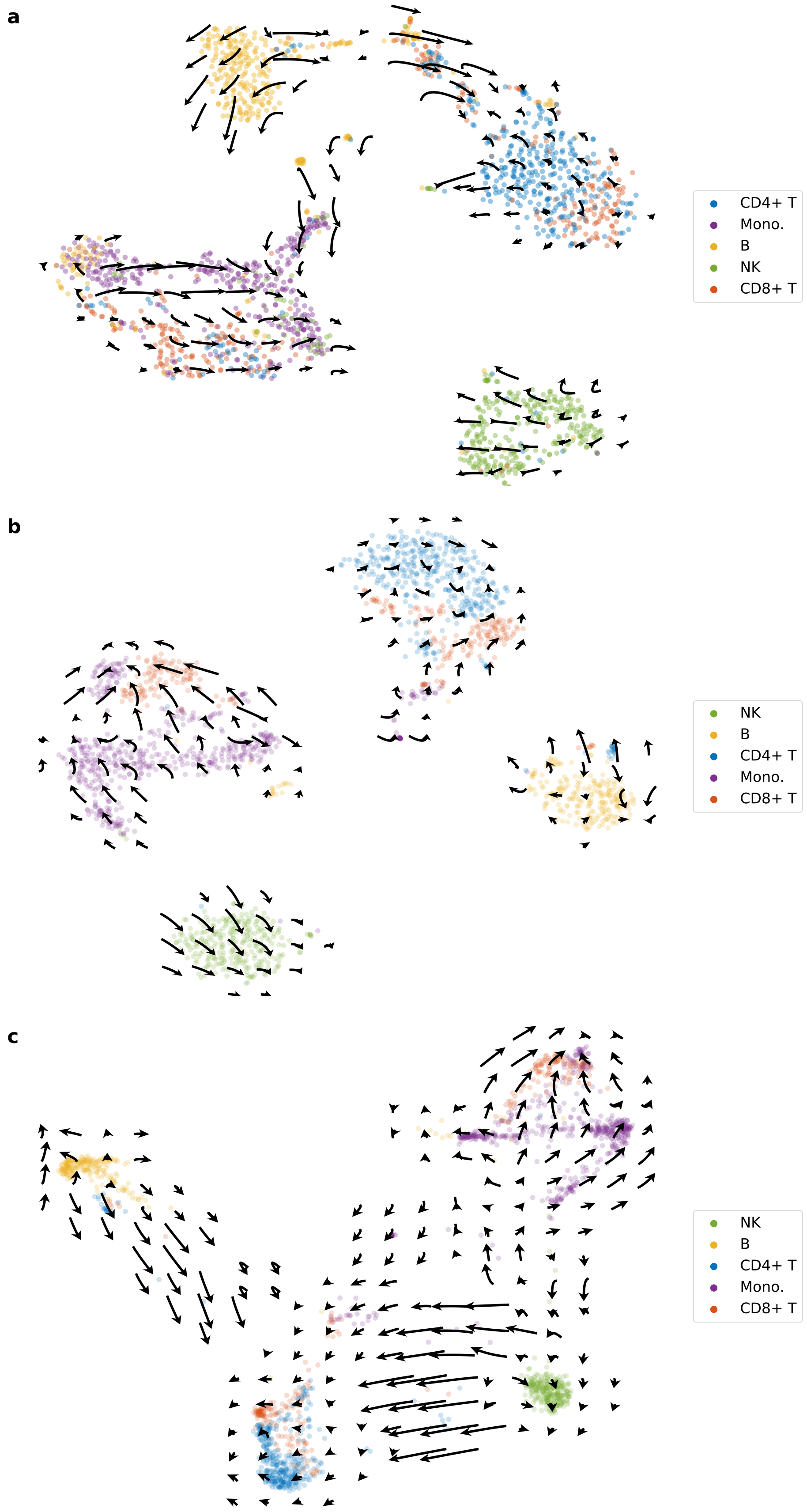

Fig. S19: Protein acceleration in CITE-seq data set projected into transcriptome-based t-SNE (a), j-SNE (b), and j-UMAP (c) embeddings.

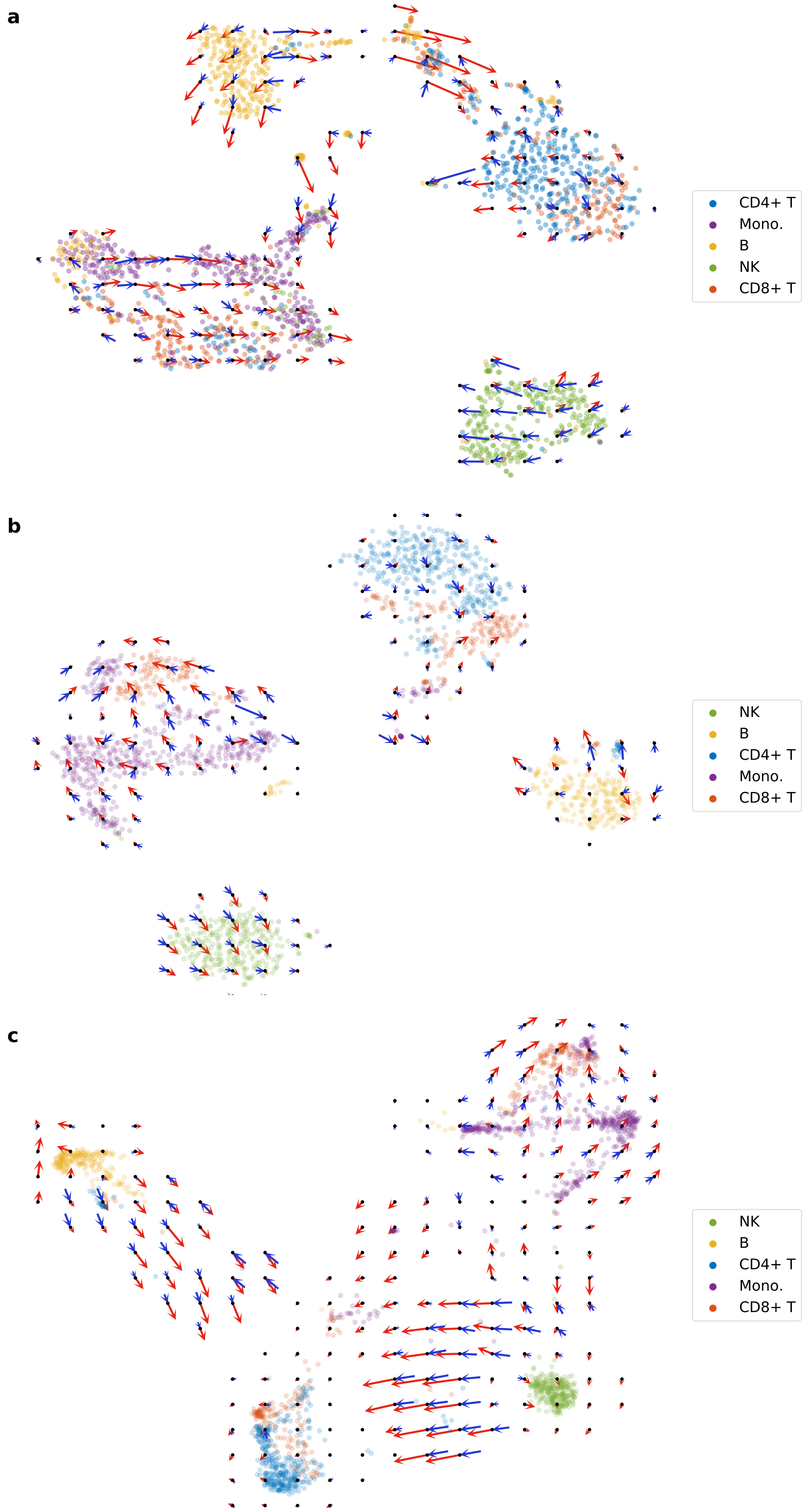

Fig. S20: CITE-seq velocity landscapes. RNA velocities (red) and protein velocities (blue) are projected into transcriptome-based t-SNE (a), j-SNE (b), and j-UMAP (c) embeddings.

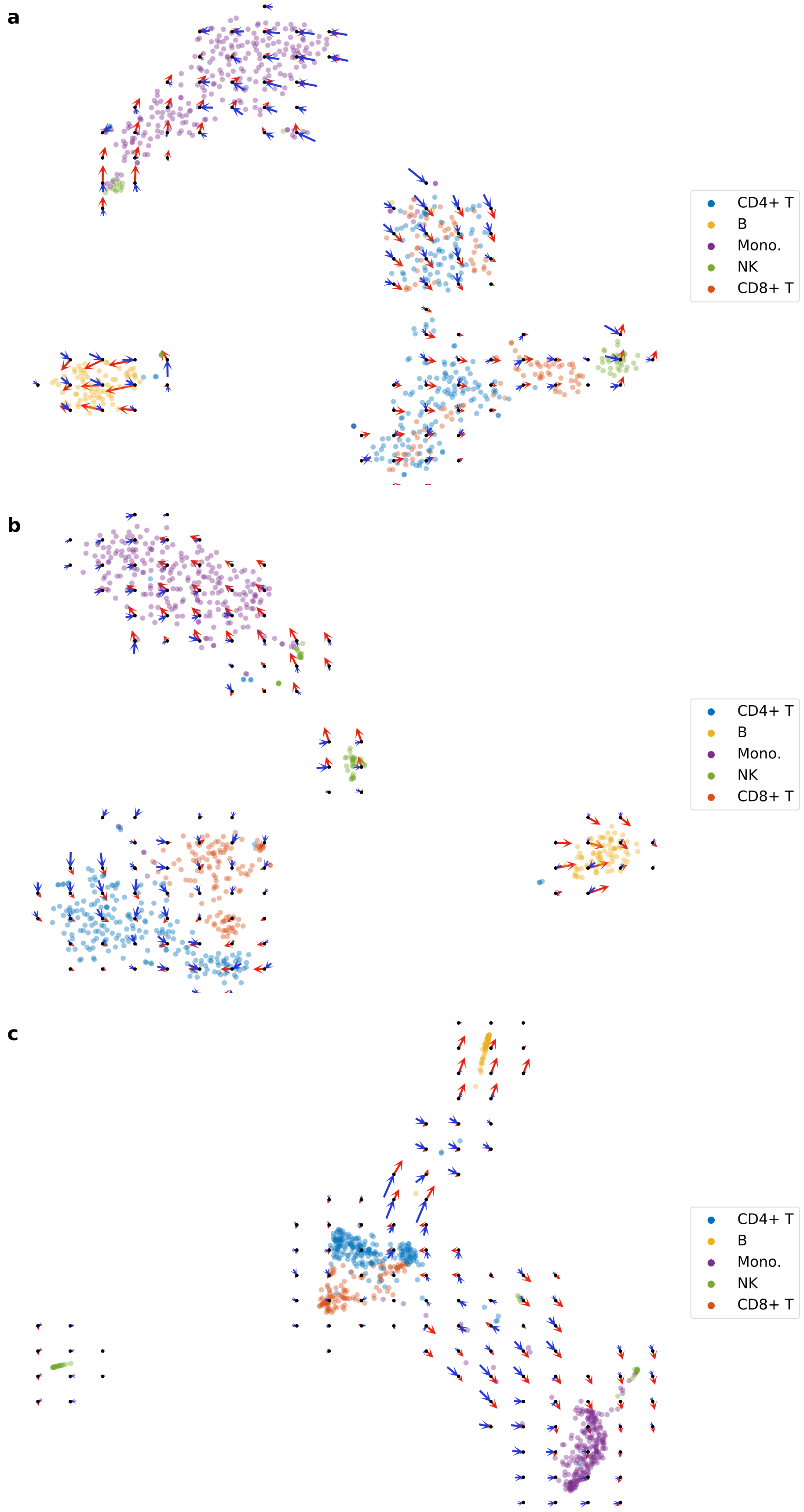

Fig. S21: 10X 1k velocity landscapes. RNA velocities (red) and protein velocities (blue) are projected into transcriptome-based t-SNE (a), j-SNE (b), and j-UMAP (c) embeddings.

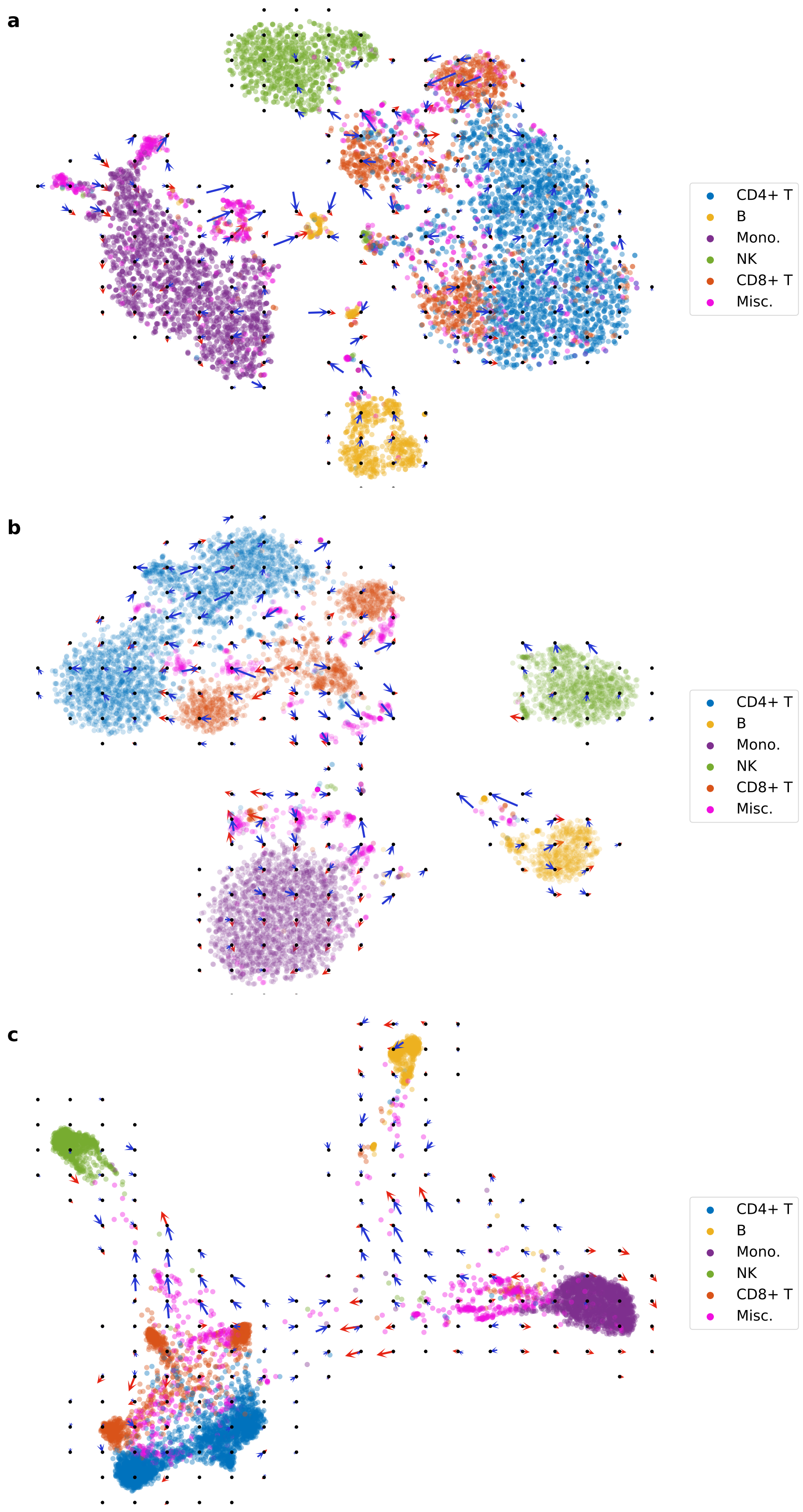

Fig. S22: 10X 10k velocity landscapes. RNA velocities (red) and protein velocities (blue) are projected into transcriptome-based t-SNE (a), j-SNE (b), and j-UMAP (c) embeddings.

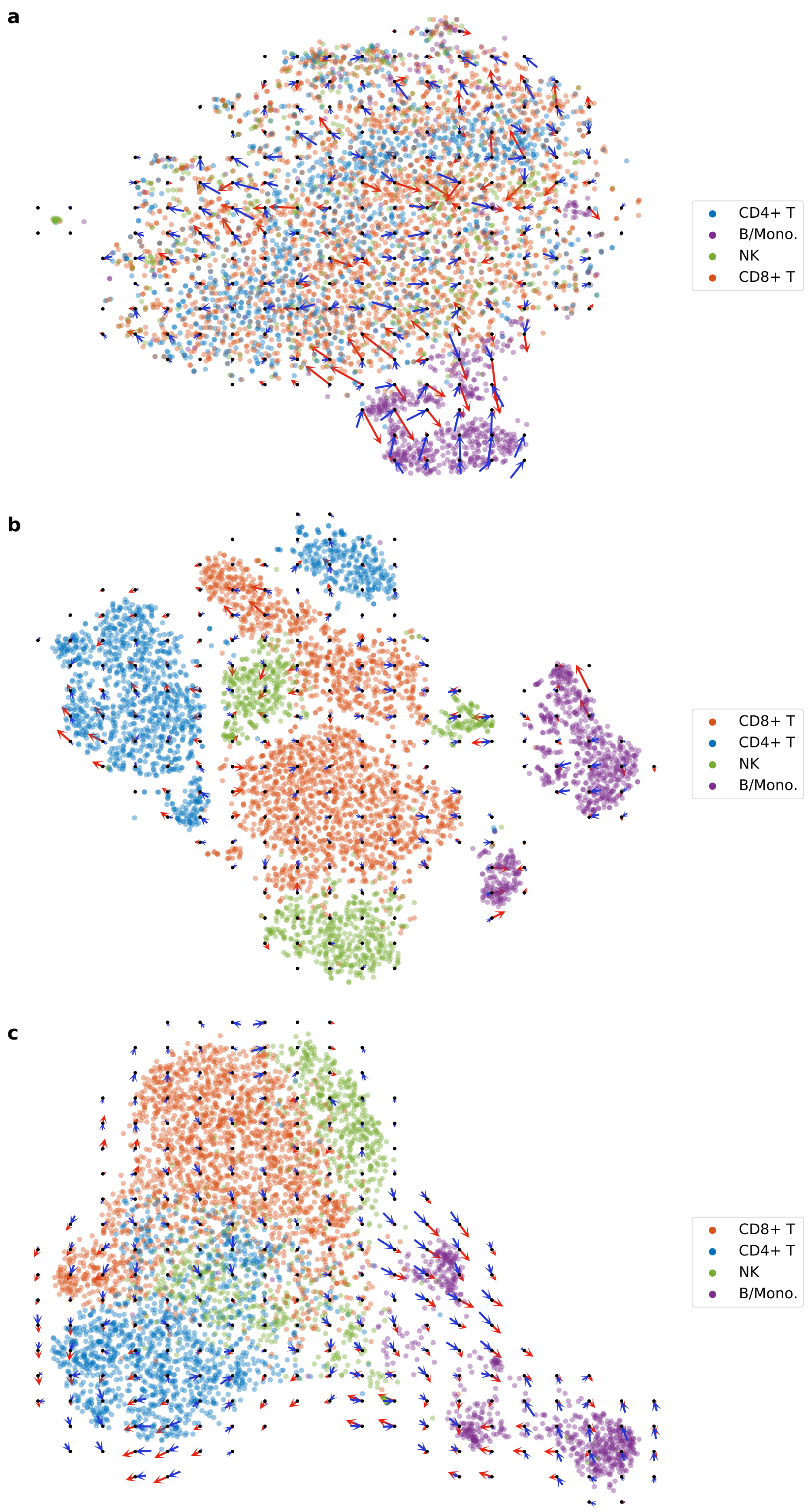

Fig. S23: ECCITE-seq (ctrl) velocity landscapes. RNA velocities (red) and protein velocities (blue) are projected into transcriptome-based t-SNE (a), j-SNE (b), and j-UMAP (c) embeddings.

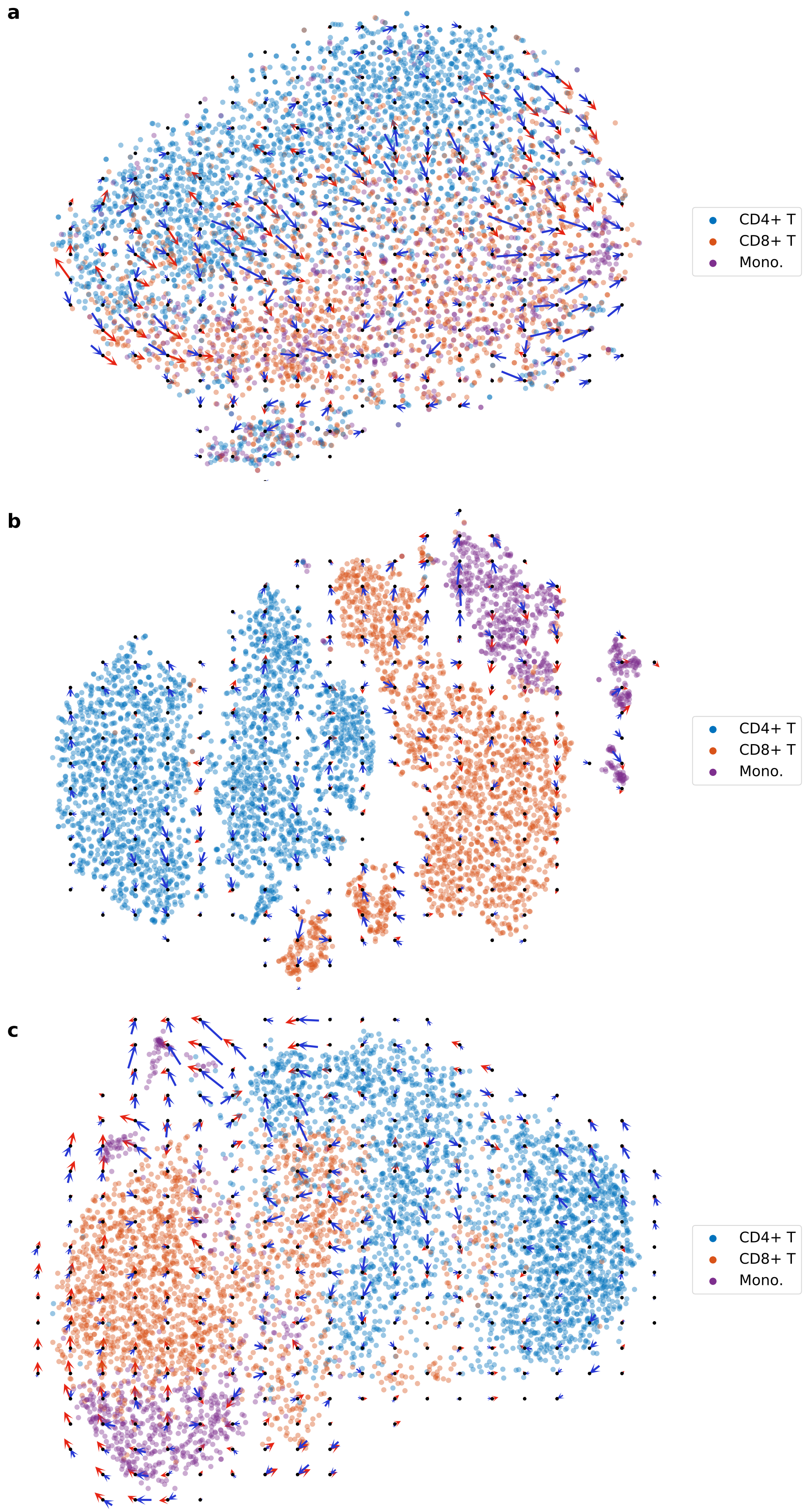

Fig. S24: ECCITE-seq (CTCL) velocity landscapes. RNA velocities (red) and protein velocities (blue) are projected into transcriptome-based t-SNE (a), j-SNE (b), and j-UMAP (c) embeddings.

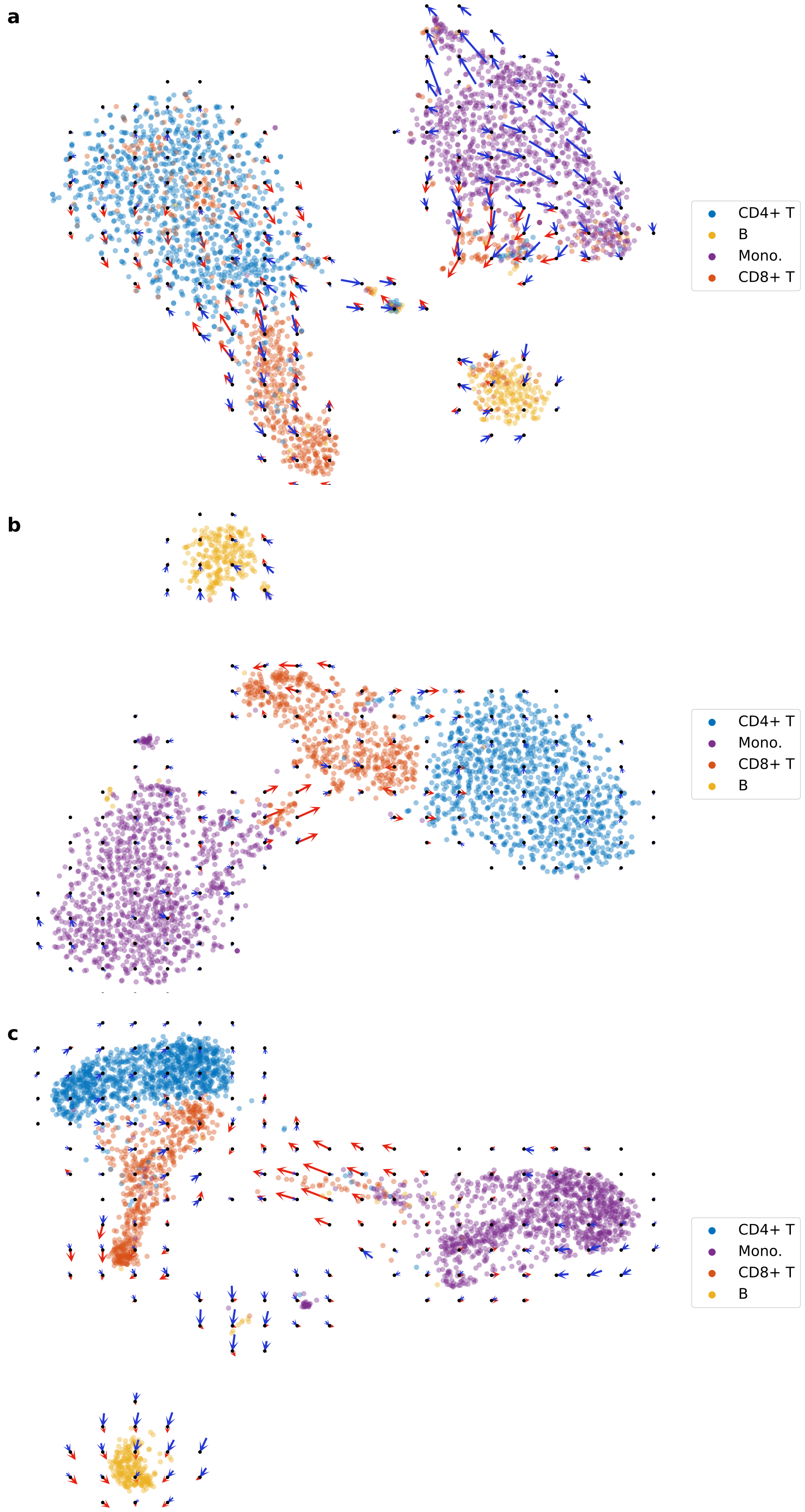

Fig. S25: REAP-seq velocity landscapes. RNA velocities (red) and protein velocities (blue) are projected into transcriptome-based t-SNE (a), j-SNE (b), and j-UMAP (c) embeddings.

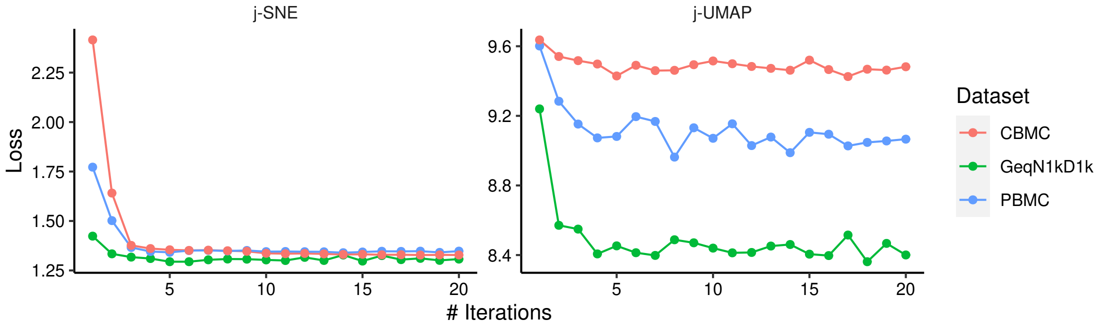

Fig. S26: Change in loss function across iterations in j-SNE and j-UMAP on 2 real data set and one simulated data set.

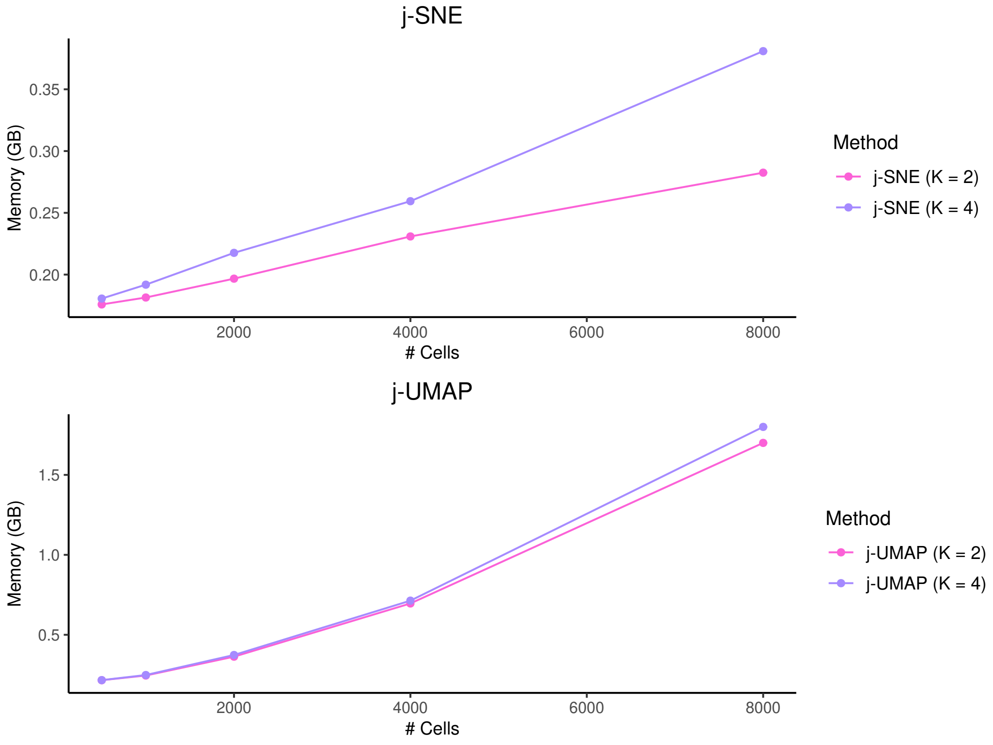

Fig. S27: Memory usage of j-SNE and j-UMAP as a function of the number of cells. The number of iterations of both methods was fixed to 10. Different size data sets were subsampled from the CBMC CITE-seq data set. Two additional modalities (K=4) were generated by duplicating and randomly shuffling the original K=2 modalities.

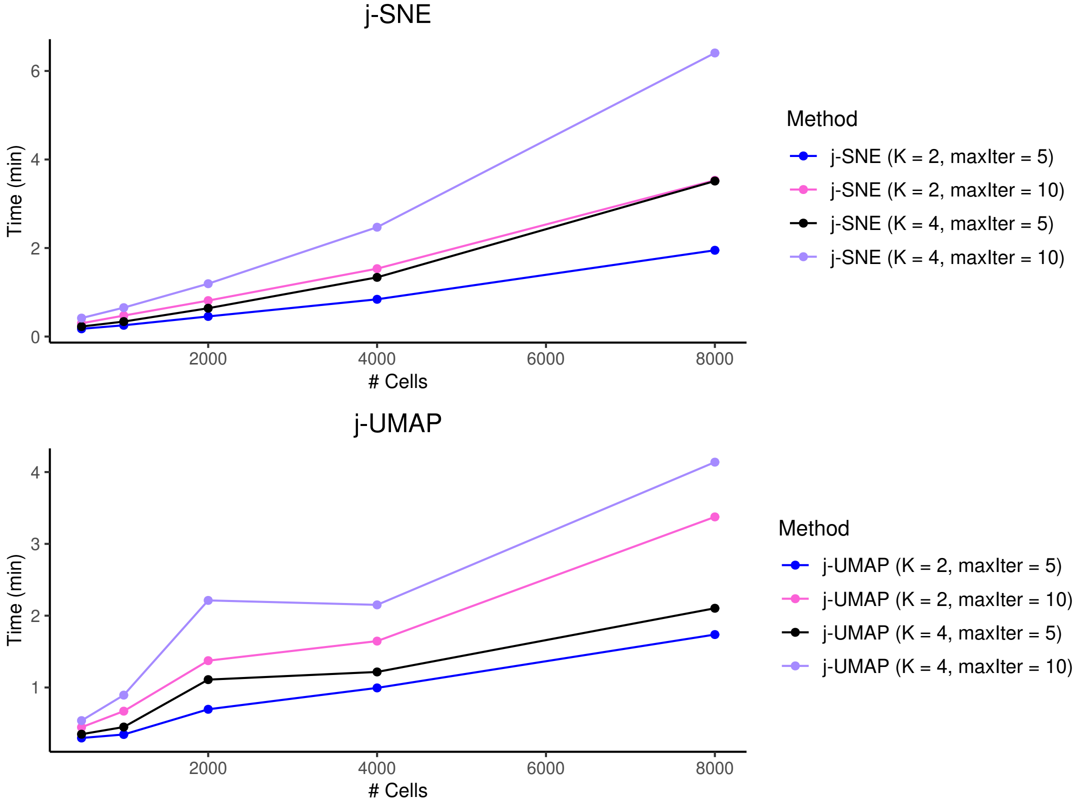

Fig. S28: CPU times of j-SNE and j-UMAP as a function of the number of cells. Different size data sets were subsampled from the CBMC CITE-seq data set. Running times on an Intel Xeon CPU at 2.30 GHz with 320 GB memory are shown using 5 or 10 iterations in both methods (controlled by parameter *maxIter*), on data sets containing K=2 and K=4 modalities. Two additional modalities were generated by duplicating and randomly shuffling the original two modalities. Note that the switching from exact to approximate nearest neighbor search in UMAP for data sets with more than $3,000$ cells slowed the increase in running time of UMAP, or even decreased it, from $2,000$ and $4,000$ cells.

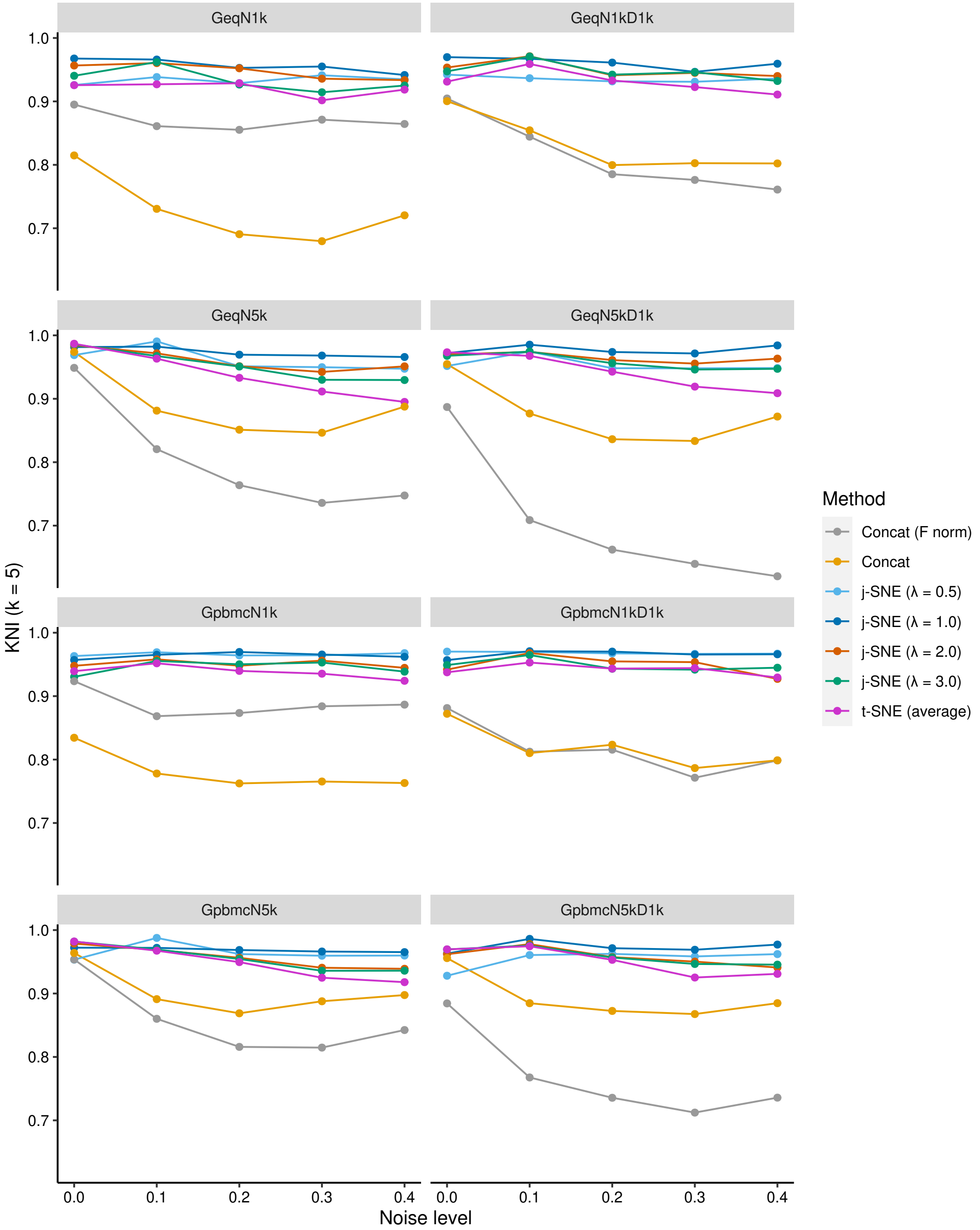

Fig. S29: KNI values (k=5) of embeddings computed by j-SNE and alternative methods on eight simulated data sets. Values are shown as a function of noise for different regularization coefficient λ used in j-SNE. Conventional t-SNE is run for uniform weights assigned to each modality ($\alpha_i = 1/3$) (t-SNE (average)), or on concatenated modalities (Concat) that are optionally normalized by the Frobenius norm (Concat (F norm)).

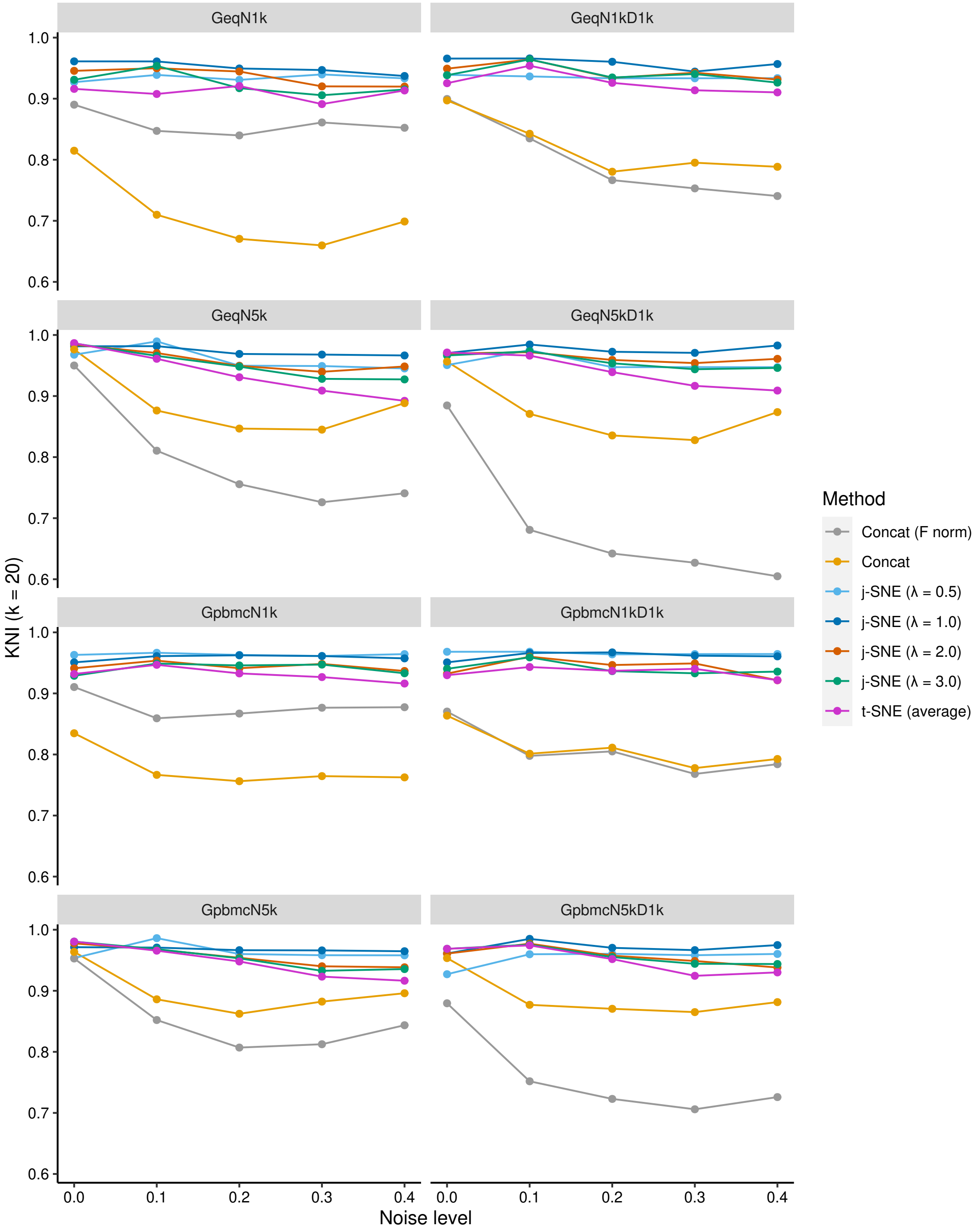

Fig. S30: KNI values (k=20) of embeddings computed by j-SNE and alternative methods on eight simulated data sets. Values are shown as a function of noise for different regularization coefficient λ used in j-SNE. Conventional t-SNE is run for uniform weights assigned to each modality ($\alpha_i = 1/3$) (t-SNE (average)), or on concatenated modalities (Concat) that are optionally normalized by the Frobenius norm (Concat (F norm)).

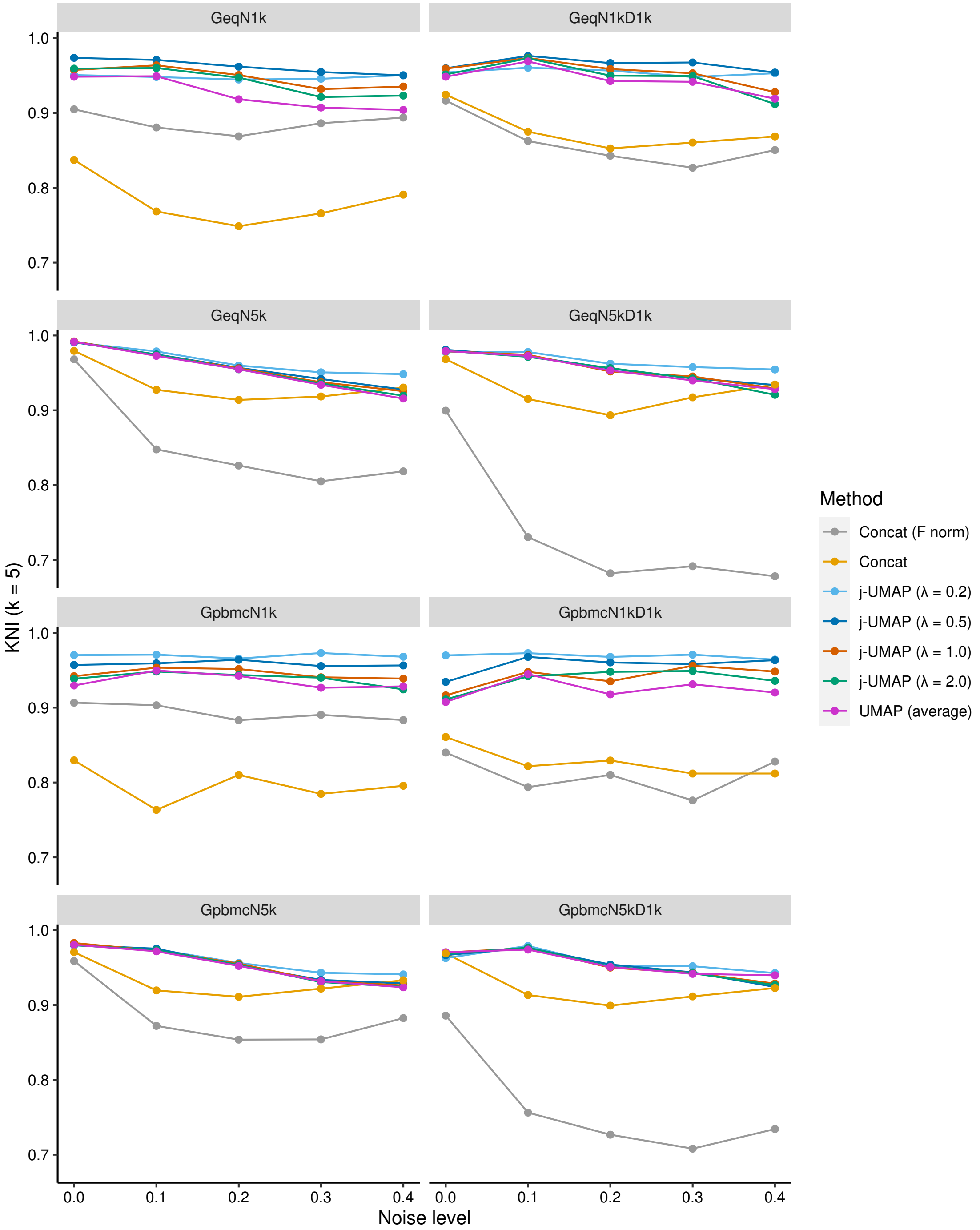

Fig. S31: KNI values (k=5) of embeddings computed by j-UMAP and alternative methods on eight simulated data sets. Values are shown as a function of noise for different regularization coefficient λ used in j-UMAP. Conventional UMAP is run for uniform weights assigned to each modality ($\alpha_i = 1/3$) (UMAP (average)), or on concatenated modalities (Concat) that are optionally normalized by the Frobenius norm (Concat (F norm)).

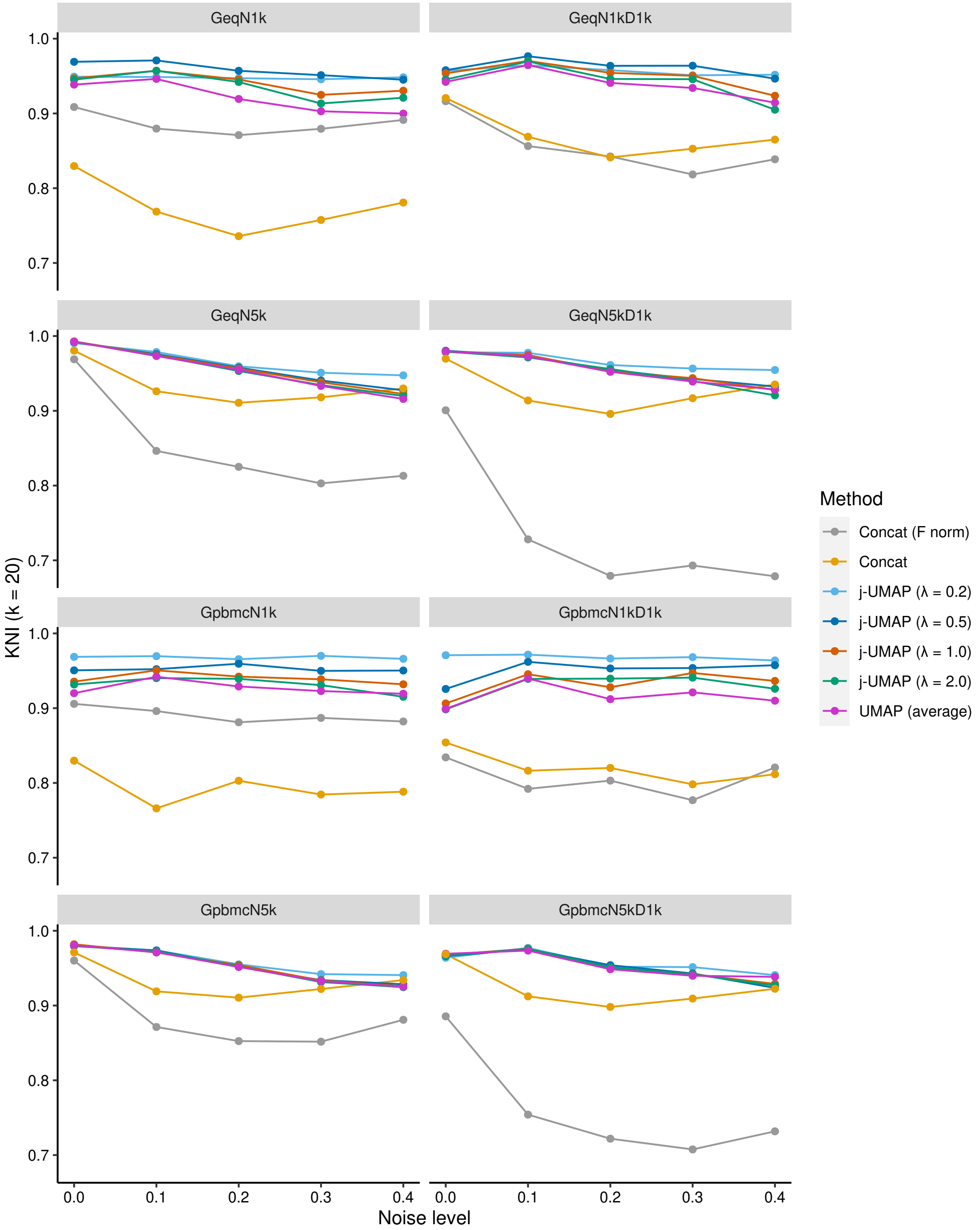

Fig. S32: KNI values (k=20) of embeddings computed by j-UMAP and alternative methods on eight simulated data sets. Values are shown as a function of noise for different regularization coefficient λ used in j-UMAP. Conventional UMAP is run for uniform weights assigned to each modality ($\alpha_i = 1/3$) (UMAP (average)), or on concatenated modalities (Concat) that are optionally normalized by the Frobenius norm (Concat (F norm)).

References

[1] van der Maaten, L., Hinton, G.: Visualizing data using t-sne. Journal of Machine Learning Research **9**(Nov), 2579–2605 (2008)

[2] van der Maaten, L.: Accelerating t-sne using tree-based algorithms. Journal of Machine Learning Research **15**(93), 3221–3245 (2014)

[3] Barnes, J., Hut, P.: A hierarchical o(n log n) force-calculation algorithm. Nature **324**(6096), 446–449 (1986). doi:10.1038/324446a0

[4] Böhm, J.N., Berens, P., Kobak, D.: A unifying perspective on neighbor embeddings along the attraction-repulsion spectrum. ArXiv e-prints at https://arxiv.org/abs/2007.08902 (2020)
