# Supplementary material for: A generalization of t-SNE and UMAP to single-cell multimodal omics
Source: Genome Biol. 2021 May 3;22:130. doi: 10.1186/s13059-021-02356-5 (PMC8091681; doi:10.1186/s13059-021-02356-5)
Supplement: Supplementary file 2 — Additional file 2 Review history [file 13059_2021_2356_MOESM2_ESM.docx]

Review History

**First round of review**

**Reviewer 1**

**Are you able to assess all statistics in the manuscript, including the appropriateness of statistical tests used?**

Yes, and I have assessed the statistics in my report.

**Comments to author:**

The paper by Do & Canzar suggests a generalization of t-SNE and UMAP for multimodal datasets and applies the resulting j-SNE and j-UMAP to the single-cell multi-omic data. t-SNE and UMAP are two very popular algorithms for visualizing single-cell data. As single-cell multi-omic data become more widespread, a joint visualization of several modalities becomes an important computational challenge.

I believe this paper addresses a relevant and timely issue. The central idea (using a regularization term that effectively penalizes noisy modalities because their KL term cannot be made small) is original and smart. At the same time, I have some reservations that I think would require at least a major revision. If the authors can address these reservations, then this paper could I think eventually be accepted to Genome Biology.

MAJOR ISSUES

* The authors stress that the weights (alpha) are optimized by the algotithm and can "effectively suppress the noise" by decreasing the weight for the noisy modality. But the noise weight is 0.19 which is arguably not very low. I have two concerns: (i) does it actually matter that the weight is not 1/3? What if the authors run only one single iteration of their method, which is basically running t-SNE on the average affinity matrix? If the results are similarly good to what is shown in the paper, then the entire method reduces to an extremely simple averaging of affinities. (Which could be implemented in libraries like openTSNE in basically one line of code.) So *if* there little practical difference between optimizing alphas and not optimizing them, this would make the method seem quite a bit less impressive. As I said above, the main novelty of the method, in my mind, lies in the idea that the weights can be optimized. The authors really need to show that it is actually beneficial in practice.

* Continuing, (ii) 0.19 isn't very low. It could presumably be made lower by increasing lambda. The authors say that lambda was fixed to 3 in all experiments, but do not justify this choice. I would like to see some experiments showing the effect of lambda and some discussion of why 3 is a good choice. In particular when one modality is pure noise, shouldn't one want to set lambda to some value that would drive that alpha weight much closer to zero? What lambda is needed for that? Would it make sense to always use such a lambda value? What are the trade-offs here?

* Another concern is the comparison to the naive concatenation approach. Clearly the t-SNE results after concatenation will strongly depend on relative norms of different modalities. If the noise modality has large values or high dimensionality, it will dominate the Euclidean distances, and yield a meaningless embedding. I have a feeling that this comparison is somewhat of a strawman. A more reasonable approach would be to normalize each modality before concatenation, e.g. by dividing each modality by the Frobenius norm of its data matrix (but other normalization choices are also possible). I'd much rather like to see a comparison of j-SNE with such a normalized concatenation approach. Hopefully, j-SNE will still outperform this approach. But if it does not, this again would make this work less impactful. For an example of such a normalized concatenation in a recent work, see e.g. Fig 3c in https://www.nature.com/articles/s41586-020-2907-3, but I am sure there are other examples.

MEDIUM ISSUES

* The Results section contains zero information on how the method works. I appreciate that there is not much space in the Short Report format, but I would still suggest to start the Results with a brief paragraph on how j-SNE/j-UMAP work. I would even strongly suggest to include at least one formula, e.g. Equation 6 from the suppl methods. And have a few sentences to explain the key idea.

* page 4, lines 47-56: This description should mention that your algorithm iteratively reruns t-SNE multiple times. How many times are needed for convergence? This is not mentioned anywhere. It would make sense to say that your algothim needs e.g. 10 times longer than t-SNE because it runs t-SNE optimization 10 times. Or something like that.

* Your j-SNE implementation is based on sklearn, but it should not be difficult to implement the same based on openTSNE, which is much faster (it's a FIt-SNE reimplementation), better supported, and has a modular structure that would be perfect for j-SNE implementation. In fact, it seems that j-SNE could be implemented strictly on top of openTSNE, without modifying the openTSNE code itself.

* Table S2: Why is ARI so low in several rows (below 0.1!) while KNI remains super high? This is very confusing, and makes me suspect that ARI was not a very meaningful metric. Please convince the reader otherwise.

MINOR ISSUES

* The Results section starts with the experiment where one modality was shuffled, which initially looks like an odd choice. Maybe you could make the expectation clearer in the beginning here and say something about a "toy experiment" or a "controlled setting" or something like that. You do say "as a proof concept", but I was still confused on the first reading on why you spend so much time talking about shuffled data.

* page 2, line 62: report the weights in the text!

* page 3, line 17: "benefit from their mutual dependence" -- unclear formulation. Dependence in what sense?

* page 4, lines 47-56: FIt-SNE is linear in the same sense as UMAP is linear. It's a bit strange to say that UMAP is O(n^1.14) but FIt-SNE is linear.

* page 5, line 52: "reveal relationships" -- unclear formulation. Does j-SNE reveal relationships betweem modalities? I'd say it uses them together but does not study *relationships* between them,

* line 56: "compared"

* Suppl Materials, page 4: it's very hard to follow the formulas because you have $a$ and $\alpha$. Can you replace $a$ with $b$?

* last sentence before "Generalizing UMAP to multimodal data": how many iterations are needed in practice?

* page 5: "analog"->"analogous"

* page 5: could be worth saying that (1-p_ij) in Eq 12 is approximated by 1 in UMAP

* page 5, last sentence: very unclear. What does it mean? Please be more specific.

* page 5: UMAP does not really optimize Eq 12 because it uses negative sampling, and there are claims in the literature that this has a drastic effect: https://arxiv.org/abs/2007.08902. I guess the authors here tweaked the existing UMAP code, so probably also are using negative sampling. I think it is worth mentionining here explicitly.

**Reviewer 2**

**Are you able to assess all statistics in the manuscript, including the appropriateness of statistical tests used?**

Yes, and I have assessed the statistics in my report.

**Comments to author:**

In this work, Do and Canzar propose extensions of t-SNE and UMAP for the joint visualization of single-cell multi-modal data. These are useful extensions for dealing with single-cell data with multiple modalities. However, we feel a more comprehensive evaluation of the proposed methods is needed to establish the utility, robustness, and efficiency of the extensions. We have the following specific comments:

1. The simulation of noise in data is interesting. However, we feel the evaluation is not as comprehensive. For example, does different amount of data noise impact on the visualization results differently? How much noise can the method tolerate and when would it break? These detailed assessments should be conducted to fully establish the utility and robustness of the extensions.

2. Related to above, how meaningful and robust are the learned weights? Would the learned weight of a modality increase as the amount of the noise decrease in that modality and vice versa? Also, how stable are the estimates of the weights of modalities and how much data is required to have a stable estimate?

3. The authors noted that "In all experiments, we set the regularization parameter "lambda" to 3 for j-SNE and to 1 for j-UMAP". We wonder how these values are determined? How sensitivity they are? And how would the j-SNE and j-UMAP results changes as lambda vary?

4. The author introduced k-nearest neighbor index (KNI) for performance assessment. This is an interesting metric and is independent from clustering which are often required for assessment using a metric like ARI. However, we feel the author should investigate the characteristics of this metric with respect to the embedding results to establish its utility in assessment. At the moment, it is not clear how informative and sensitive this metric is towards "good" and "bad" results from t-SNE, j-SNE, UMAP, and j-UMAP. The choice of k=10 is another hyperparameter that should be investigated. How much impact does this value have on the evaluation results?

5. Computation time and memory usage: The last paragraph of results about the computation time is vague. Given the increasing size of single-cell omics data, the runtime complexity and the memory usage of j-SNE and j-UMAP should be computed and plotted against the number of cells and the number of modalities. The details of the computer used for assessing computation time should also be reported.

6. We also wonder if the extensions are applicable in the situation that the different modalities are not measured from the same cells? This scenario is common given that not all modalities can be measured simultaneously from the same "matched" cells and we often obtain different modalities from different samples of the same cell populations. Any extension toward this will significantly broaden the utility of the methods.

7. The authors noted in the second last paragraph that "We used standard preprocessing of the input data including log-transformation of the expression matrix followed by principal component analysis (PCA) for dimensionality reduction to 20 principle components". Please clarify whether the j-SNE and j-UMAP were applied after PCA reduction of the data to 20 PCs? t-SNE and UMAP are often used as alternative to PCA. Assuming the authors used PCA prior to the application of t-SNE, j-SNE, UMAP, and j-UMAP, would the above findings hold if we were to apply these methods directly to the log-transformed data and without PCA dimension reduction? And also, how would the computation time and memory usage change?

**Reviewer 3**

**Are you able to assess all statistics in the manuscript, including the appropriateness of statistical tests used?**

Yes, and I have assessed the statistics in my report.

**Comments to author:**

This manuscript presents joint t-SNE & UMAP to find a low-dimensional representation of single cell data. The major advance is that the proposed joint embedding methods use linear combination of the original objective function (KL-divergence and cross entropy, respectively) and an evenly distributed regularization (penalty for the coefficients alpha-k). An iterative optimization method is proposed to find the solution.

1) As a method paper, the current advance was limited. But from the view of a software paper, this tool should be useful for the multi-model single cell analysis.

2) If possible, a R version should be also developed (especially for a sofware-like paper).

3) Seurat also presents a novel method to integraive analyze multi-model single cell data. The proposed methods should compare with it.

4) In several cases, the joint embedding methods did not obviously show better results than single-model data (such as Figures S4 & S6). In my opinion, before the joint analysis, we should carefully check the consistency between the single-model results. Joint is not always better than single (it is a general suggestion for joint analysis..)

**Authors Response**

**Point-by-point responses to the reviewers’ comments:**

Reviewer #1

The paper by Do & Canzar suggests a generalization of t-SNE and UMAP for multimodal datasets and applies the resulting j-SNE and j-UMAP to the single-cell multi-omic data. t-SNE and UMAP are two very popular algorithms for visualizing single-cell data. As single-cell multi-omic data become more widespread, a joint visualization of several modalities becomes an important computational challenge.

I believe this paper addresses a relevant and timely issue. The central idea (using a regularization term that effectively penalizes noisy modalities because their KL term cannot be made small) is original and smart. At the same time, I have some reservations that I think would require at least a major revision. If the authors can address these reservations, then this paper could I think eventually be accepted to Genome Biology.

*We thank the reviewer for the positive assessment of our work.*

MAJOR ISSUES

The authors stress that the weights (alpha) are optimized by the algotithm and can "effectively suppress the noise" by decreasing the weight for the noisy modality. But the noise weight is 0.19 which is arguably not very low. I have two concerns: (i) does it actually matter that the weight is not 1/3? What if the authors run only one single iteration of their method, which is basically running t-SNE on the average affinity matrix? If the results are similarly good to what is shown in the paper, then the entire method reduces to an extremely simple averaging of affinities. (Which could be implemented in libraries like openTSNE in basically one line of code.) So *if* there little practical difference between optimizing alphas and not optimizing them, this would make the method seem quite a bit less impressive. As I said above, the main novelty of the method, in my mind, lies in the idea that the weights can be optimized. The authors really need to show that it is actually beneficial in practice.

*We thank the reviewer for this valuable suggestion. We have devised a simulation study to examine the effect of learned weights in j-SNE and j-UMAP. We generated 8 synthetic data sets using parameters learned from real CITE-seq data (see description in text) and compared the performance of our method to the averaging approach mentioned by this reviewer using metrics KNI and the Silhouette score (see response to ARI vs KNI comment below). Learned weights yielded a better separation of cell types than the simple averaging method using uniform weights (Supp Figs S7-S10). As expected, this benefit was more pronounced for smaller values of lambda that allow the weights to deviate from a uniform setting (Supp Figs S3-S5). For data sets containing 5000 cells the advantage of learned weights became more apparent in j-SNE with increasing levels of noise, again in particular for smaller values of lambda (Supp Figs S7 and S9). Consistently, on these data sets the optimal weight assigned to the noise modality by j-SNE dropped the most with increasing levels of noise (Supp Fig S3). For j-UMAP, on the other hand, computed weights were close to uniform for lambda >= 0.5 on data sets GpbmcN5k and GpbmcN5kD1k (Supp Fig S4) and thus not surprisingly yielded embeddings with similar accuracy as the uniform averaging approach on these data sets (Supp Fig S8 and S10).*

Continuing, (ii) 0.19 isn't very low. It could presumably be made lower by increasing lambda. The authors say that lambda was fixed to 3 in all experiments, but do not justify this choice. I would like to see some experiments showing the effect of lambda and some discussion of why 3 is a good choice. In particular when one modality is pure noise, shouldn't one want to set lambda to some value that would drive that alpha weight much closer to zero? What lambda is needed for that? Would it make sense to always use such a lambda value? What are the trade-offs here?

*We appreciate the reviewer’s valid concern. Indeed, the weight adjustment to increasing noise levels is more pronounced for smaller values of lambda (Supp Figs S3,S4). Supp Fig S5 shows that lambda values close to zero do drive the weight associated with the perturbed modality to zero. However, too small penalties will result in the selection of only the one most informative modality (weights 1,0,0) and therefore no longer benefit from a combination of multiple (informative) modalities. We have formalized this observation at the end of Section “Generalizing t-SNE to multimodal data” in the Supplement.*

*According to the results of our simulation study, lambda=3 is a rather conservative choice that was mostly guided by visual inspection of known cell types. Similar to the choice of resolution when clustering cells, or the perplexity value used in conventional t-SNE, we believe that visualizing cells based on varying values of lambda can provide different yet meaningful representation of cells based on varying contributions of different types of molecules. In general, higher expected levels of noise would suggest lower values of lambda to allow non-uniform weights to counteract noisy modalities, which we suggested in the Conclusion section.*

Another concern is the comparison to the naive concatenation approach. Clearly the t-SNE results after concatenation will strongly depend on relative norms of different modalities. If the noise modality has large values or high dimensionality, it will dominate the Euclidean distances, and yield a meaningless embedding. I have a feeling that this comparison is somewhat of a strawman. A more reasonable approach would be to normalize each modality before concatenation, e.g. by dividing each modality by the Frobenius norm of its data matrix (but other normalization choices are also possible). I'd much rather like to see a comparison of j-SNE with such a normalized concatenation approach. Hopefully, j-SNE will still outperform this approach. But if it does not, this again would make this work less impactful. For an example of such a normalized concatenation in a recent work, see e.g. Fig 3c in https://www.nature.com/articles/s41586-020-2907-3, but I am sure there are other examples.

*We appreciate this valid concern. In our simulation study we have additionally compared our method to the normalized concatenation approach suggested by this reviewer. While this normalized version of the concatenation approach works reasonably well on some data sets containing 1000 cells, it’s performance varied substantially between data sets and yielded even less accurate embeddings than the variant without normalization on data sets with 5000 cells. In addition to the Frobenius norm, we have also experimented with the maximum norm which however yielded substantially less accurate embeddings on all data sets (not shown).*

MEDIUM ISSUES

The Results section contains zero information on how the method works. I appreciate that there is not much space in the Short Report format, but I would still suggest to start the Results with a brief paragraph on how j-SNE/j-UMAP work. I would even strongly suggest to include at least one formula, e.g. Equation 6 from the suppl methods. And have a few sentences to explain the key idea.

*Thank you for the suggestions. We have moved the high-level methods description (from Methods) to the beginning of the Results section and included equation 6.*

page 4, lines 47-56: This description should mention that your algorithm iteratively reruns t-SNE multiple times. How many times are needed for convergence? This is not mentioned anywhere. It would make sense to say that your algothim needs e.g. 10 times longer than t-SNE because it runs t-SNE optimization 10 times. Or something like that.

*In Supplementary Figure S26 we now show the change in the optimized objective across iterations. After only a few iterations (<=5) the objective converges towards its final value. We believe the lower stability in UMAP loss might be due to internal approximations (e.g. negative sampling). We have more clearly pointed out the repeated calculation of (conventional) t-SNE and UMAP in our iterative scheme (same paragraph). However, we believe the running time cannot be phrased as a small multiple of the time required by conventional t-SNE. In j-SNE we compute a single embedding based on multiple modalities, whereas t-SNE would need to compute a separate embedding for each modality. In addition, later iterations typically require less time since, for example, the nearest neighbor graph does not need to be recomputed each time (compare running times for 5 and 10 iterations in Supp Fig S28).*

Your j-SNE implementation is based on sklearn, but it should not be difficult to implement the same based on openTSNE, which is much faster (it's a FIt-SNE reimplementation), better supported, and has a modular structure that would be perfect for j-SNE implementation. In fact, it seems that j-SNE could be implemented strictly on top of openTSNE, without modifying the openTSNE code itself.

*We thank the reviewer for this valuable suggestion. In this revision we focused on additional experiments demonstrating the benefit of our approach. We are planning to integrate JVis with scanpy and are currently exploring compatibility with openTSNE in this context.*

Table S2: Why is ARI so low in several rows (below 0.1!) while KNI remains super high? This is very confusing, and makes me suspect that ARI was not a very meaningful metric. Please convince the reader otherwise.

*We thank the reviewer for pointing out the lack of clarity regarding the choice of performance metrics. KNI indeed shows generally high values in our experiments. By definition, only “boundary cells” will potentially contribute towards a value strictly smaller than 1. They may be outweighed by “inner cells” (especially for abundant cell types) that are more likely to have homogenous neighborhoods and thus will contribute to a KNI value of 1. The ARI, on the other hand, depends on the outcome of a clustering algorithm which might be susceptible to even small differences in the embedding. We therefore replaced the ARI by the Silhouette score, another widely used metric in cluster analysis that measures how much cell types overlap (score 0) or how well separated (score 1) they are. For this, we only rely on the ground truth labeling, no further clustering of cells is required. Together, KNI and the Silhouette score provide a consistent picture of relative method performance, but distinguish between methods (or parameters) with different resolutions.*

MINOR ISSUES

The Results section starts with the experiment where one modality was shuffled, which initially looks like an odd choice. Maybe you could make the expectation clearer in the beginning here and say something about a "toy experiment" or a "controlled setting" or something like that. You do say "as a proof concept", but I was still confused on the first reading on why you spend so much time talking about shuffled data.

*We apologize for the confusion regarding this experiment. We have added “in a toy experiment” when describing the random shuffling of gene expression measurements. In addition, we have replaced the experiment based on a simplistic randomly generated third modality by a systematic simulation study in which we explore the effect of noise and selection of parameters on method performance.*

page 2, line 62: report the weights in the text!

*We now systematically explore the change in weights calculated by our method for varying algorithm and instance parameters and in particular show their dependence on the level of noise and selection of lambda in Supp Figs S3-S5.*

page 3, line 17: "benefit from their mutual dependence" -- unclear formulation. Dependence in what sense?

*We apologize for the confusion. We have removed this statement.*

page 4, lines 47-56: FIt-SNE is linear in the same sense as UMAP is linear. It's a bit strange to say that UMAP is O(n^1.14) but FIt-SNE is linear.

*We apologize for possibly misunderstanding this comment, but with this we were referring to the empirical complexity of UMAP given in the original work. And according to [22] the theoretical complexity of FIt-SNE has a linear dependence on the number of points. Nevertheless, we have rephrased the statement about linear complexity.*

page 5, line 52: "reveal relationships" -- unclear formulation. Does j-SNE reveal relationships betweem modalities? I'd say it uses them together but does not study *relationships* between them,

*We believe that downstream analyses of joint embeddings may help to reveal relationships between different types of molecules. j-SNE and j-UMAP themselves, however, try to exploit such relationships, as this reviewer correctly pointed out. We have rephrased this statement.*

line 56: "compared"

*Thank you, we have corrected this error.*

Suppl Materials, page 4: it's very hard to follow the formulas because you have $a$ and $\alpha$. Can you replace $a$ with $b$?

*Thank you for pointing this out. We have replaced a with b.*

last sentence before "Generalizing UMAP to multimodal data": how many iterations are needed in practice?

*We have added an analysis of the required number of iterations in Supp Fig S26. In all experiments on real data sets we have set maxIter to 10 (see Methods).*

page 5: "analog"->"analogous"

*We have corrected this error.*

page 5: could be worth saying that (1-p_ij) in Eq 12 is approximated by 1 in UMAP

*We were not able to confirm this approximation based on the original work [10]. We therefore prefer to not make this claim.*

page 5, last sentence: very unclear. What does it mean? Please be more specific.

*We apologize for the lack of clarity. We have added a formal description.*

page 5: UMAP does not really optimize Eq 12 because it uses negative sampling, and there are claims in the literature that this has a drastic effect: https://arxiv.org/abs/2007.08902. I guess the authors here tweaked the existing UMAP code, so probably also are using negative sampling. I think it is worth mentionining here explicitly.

*Thank you for pointing this out, we have mentioned this right below equation 12.*

Reviewer #2

In this work, Do and Canzar propose extensions of t-SNE and UMAP for the joint visualization of single-cell multi-modal data. These are useful extensions for dealing with single-cell data with multiple modalities.

*We thank the reviewer for the positive assessment of our work.*

However, we feel a more comprehensive evaluation of the proposed methods is needed to establish the utility, robustness, and efficiency of the extensions. We have the following specific comments:

1. The simulation of noise in data is interesting. However, we feel the evaluation is not as comprehensive. For example, does different amount of data noise impact on the visualization results differently? How much noise can the method tolerate and when would it break? These detailed assessments should be conducted to fully establish the utility and robustness of the extensions.

*We appreciate the reviewer’s very valuable feedback. We have added a simulation study to investigate (among others) the effect of noise on the final embeddings produced by j-SNE and j-UMAP. We generated 8 synthetic data sets using parameters learned from real CITE-seq data (see description in text). For small enough penalties (lambda) assigned to non-uniform weights, the weight associated with the noise modality continuously decreased with increasing noise levels to counteract the noise (Supp Figs S3, S4). For several data sets, the accuracy of the resulting embeddings was only slightly affected even when permuting gene expression measurements between up to 40% of cells (Supp Figs S7-S10). We observed the sharpest decrease in accuracy in terms of Silhouette score for data sets containing 5000 cells with an unbalanced cell type composition (GpbmcN5k, Supp Figs S9 and S10). The benefit over concatenation-based approaches (Supp Figs S7-S10) as well as the positive effect of learned weights on the final j-SNE embedding (compare to uniform averaging scheme in Supp Figs S7 and S9), however, persist even on these data sets.*

*In practice, the user has control over the degree to which our method will make use of multiple modalities. Higher expected levels of noise would suggest lower values of lambda to allow non-uniform weights to counteract noisy modalities (see e.g. data set GeqN5K with 40% of cells swapped in Supplementary Figure S3). For values of lambda close to 0, our method will tend to pick the one most informative modality (Supp Fig S5) and thus behave similarly to traditional t-SNE and UMAP on unimodal data. We have formalized this observation at the end of Section “Generalizing t-SNE to multimodal data” in the Supplement.*

2. Related to above, how meaningful and robust are the learned weights? Would the learned weight of a modality increase as the amount of the noise decrease in that modality and vice versa? Also, how stable are the estimates of the weights of modalities and how much data is required to have a stable estimate?

*Supp Figs S3 and S4 shows the dependence of learned weights on the level of noise. As expected, the weight associated with the noise modality continuously decreased with increasing noise level, provided a small enough penalty (lambda) allowed such a deviation from uniform weights. As suggested by reviewer #1, we compared our method to an approach that uses uniform weights (here 1/3). Learned weights yielded a better separation of cell types than the simple averaging method using uniform weights (Supp Figs S7-S10) and this benefit was more pronounced for smaller values of lambda that allow the weights to deviate from a uniform setting (Supp Figs S3-S5). For data sets containing 5000 cells the advantage of learned weights became more apparent in j-SNE with increasing levels of noise, again in particular for smaller values of lambda (Supp Figs S7 and S9). Consistently, on these data sets the optimal weight assigned to the noise modality by j-SNE dropped the most with increasing levels of noise (Supp Fig S3). For j-UMAP, on the other hand, computed weights were close to uniform for lambda >= 0.5 on data sets GpbmcN5k and GpbmcN5kD1k (Supp Fig S4) and thus not surprisingly yielded embeddings with similar accuracy as the uniform averaging approach on these data sets (Supp Fig S8 and S10).*

*Supp Fig S6 shows mRNA and ADT weights computed for random subsamples of cells of different size. The variance across 10 random trials is close to zero demonstrating consistent optimal weights for different equal-size subsamples of the data. Even when eliminating as much as half of the data the computed weights remain stable. We point out, however, that even when weights differ for random subsamples of the data, they represent best possible (according to our optimized objective) solutions for the subsampled data rather than inaccurate estimates of “true” weights with respect to the full data.*

3. The authors noted that "In all experiments, we set the regularization parameter "lambda" to 3 for j-SNE and to 1 for j-UMAP". We wonder how these values are determined? How sensitivity they are? And how would the j-SNE and j-UMAP results changes as lambda vary?

*We apologize for the lack of clarity in the choice of lambda. We have added Supp Figs S3 and S4 in which we examine the impact of noise on the weights for different values of lambda. As expected, the weight adjustment to increasing noise levels is more pronounced for smaller values of lambda, which generally improved the accuracy of the resulting embedding (see previous answer). For a fixed noise level (0.4), Supp Fig S5 shows that lambda values close to zero drive the weight associated with the perturbed modality to zero. However, too small penalties result in the selection of only the one most informative modality (weights 1,0,0, see formalization at the end of Section “Generalizing t-SNE to multimodal data” in Supplementary Methods) and therefore no longer benefit from a combination of multiple (informative) modalities.*

*According to the results of our simulation study, lambda=3 is a rather conservative choice that was mostly guided by visual inspection of known cell types. Similar to the choice of resolution when clustering cells, or the perplexity value used in conventional t-SNE, we believe that visualizing cells based on varying values of lambda can provide different yet meaningful representation of cells based on varying contributions of different types of molecules. In general, higher expected levels of noise would suggest lower values of lambda to allow non-uniform weights to counteract noisy modalities, which we suggested in the Conclusion section.*

4. The author introduced k-nearest neighbor index (KNI) for performance assessment. This is an interesting metric and is independent from clustering which are often required for assessment using a metric like ARI. However, we feel the author should investigate the characteristics of this metric with respect to the embedding results to establish its utility in assessment. At the moment, it is not clear how informative and sensitive this metric is towards "good" and "bad" results from t-SNE, j-SNE, UMAP, and j-UMAP. The choice of k=10 is another hyperparameter that should be investigated. How much impact does this value have on the evaluation results?

*We appreciate this valid concern. As this reviewer correctly pointed out, the ARI depends on the outcome of a clustering algorithm which might be susceptible to even small differences in the embedding. We therefore replaced the ARI by the Silhouette score, another widely used metric in cluster analysis that measures how much cell types overlap (score 0) or how well separated (score 1) they are. Similar to KNI, we only rely on the ground truth labeling, no further clustering of cells is required. Importantly, KNI and the Silhouette score provide a consistent picture of relative method performance, but distinguish between methods (or parameters) with different resolutions (see Supp Figs S7-S10). KNI shows generally high values in our simulation study for different values of lambda and across noise levels. We attribute this property to its dependence on “boundary cells” that will potentially contribute towards a value strictly smaller than 1. These may be outweighed by “inner cells” that are more likely to have homogenous neighborhoods and thus will contribute to a KNI value of 1. Nevertheless, we believe that the two metrics together can provide a more comprehensive assessment of method performance as they measure different aspects of the embedding (local neighborhoods vs global separation of cell types).*

*Supplementary Figures S29-S32 show that the results are highly consistent for different values of k used in the definition of metric KNI.*

5. Computation time and memory usage: The last paragraph of results about the computation time is vague. Given the increasing size of single-cell omics data, the runtime complexity and the memory usage of j-SNE and j-UMAP should be computed and plotted against the number of cells and the number of modalities. The details of the computer used for assessing computation time should also be reported.

*Thank you for this suggestion. In Supplementary Figures S27 and S28 we show memory usage and running times as a function of the number of cells with 2 and 4 modalities as measured by current multimodal assays. Importantly, in Supplementary Figure S26 we demonstrate that our alternating minimization scheme needs only a few (<=5) iterations to obtain solutions with a value close to the final score. We believe the lower stability in UMAP loss might be due to internal approximations (e.g. negative sampling). We provide the specifics of the computing resource in the caption of Figure S28.*

6. We also wonder if the extensions are applicable in the situation that the different modalities are not measured from the same cells? This scenario is common given that not all modalities can be measured simultaneously from the same "matched" cells and we often obtain different modalities from different samples of the same cell populations. Any extension toward this will significantly broaden the utility of the methods.

*Our model makes explicit use of the fact that different modalities were obtained from the same cell and thus cannot be applied to the scenario described by this reviewer. We believe, however, that our method will nevertheless be able to provide valuable guidance and insights in the analysis of rapidly growing numbers of multimodal data sets.*

7. The authors noted in the second last paragraph that "We used standard preprocessing of the input data including log-transformation of the expression matrix followed by principal component analysis (PCA) for dimensionality reduction to 20 principle components". Please clarify whether the j-SNE and j-UMAP were applied after PCA reduction of the data to 20 PCs? t-SNE and UMAP are often used as alternative to PCA. Assuming the authors used PCA prior to the application of t-SNE, j-SNE, UMAP, and j-UMAP, would the above findings hold if we were to apply these methods directly to the log-transformed data and without PCA dimension reduction? And also, how would the computation time and memory usage change?

*We thank the reviewer for pointing out the lack of clarity regarding the preprocessing performed in our experiments. We have rephrased this statement to more clearly describe the application of j-SNE and j-UMAP to preprocessed data by PCA. We point out that we followed current best practices [25] when using PCA as a pre-processing step for non-linear dimensionality reduction. Similarly, the Seurat pipeline and FIt-SNE [22] designed for large data sets initially run PCA. We therefore decided to apply consistent pre-processing (including PCA) on mRNA counts in all our experiments.*

Reviewer #3

This manuscript presents joint t-SNE & UMAP to find a low-dimensional representation of single cell data. The major advance is that the proposed joint embedding methods use linear combination of the original objective function (KL-divergence and cross entropy, respectively) and an evenly distributed regularization (penalty for the coefficients alpha-k). An iterative optimization method is proposed to find the solution.

1) As a method paper, the current advance was limited. But from the view of a software paper, this tool should be useful for the multi-model single cell analysis.

*We thank the reviewer for the positive assessment of our work.*

2) If possible, a R version should be also developed (especially for a sofware-like paper).

*Thank you for this suggestion. We believe that j-SNE and j-UMAP will be accessible through Python package JVis in the PyPI software repository to a large community interested in single cell analysis and methods development. It is extremely easy to use and requires only a few lines of code (see https://github.com/canzarlab/JVis-learn). Furthermore, we are planning to integrate our multimodal visualization with scanpy, a widely used Python toolkit for single-cell analysis.*

3) Seurat also presents a novel method to integraive analyze multi-model single cell data. The proposed methods should compare with it.

*While several methods have been proposed for the general task of an integrated analysis of multi-modal single cell data, including for example Seurat and MOFA+ (Argelaguet et al 2020), here we propose a method that is specifically designed for the joint visualization of 2 or more modalities. It generalizes the optimization schemes underlying widely used visualization techniques t-SNE and UMAP that are currently applied either “side-by-side” for each modality, or to a normalized concatenation of modalities as pointed out by reviewer #1 (https://www.nature.com/articles/s41586-020-2907-3,). We therefore followed the suggestions by reviewer #1 and compared our method against such a normalized concatenation approach (Supp Figs S7-S10). While this normalized concatenation approach worked reasonably well on some simulated data sets containing 1000 cells, it’s performance varied substantially between data sets and yielded even less accurate embeddings than the variant without normalization on data sets with 5000 cells.*

4) In several cases, the joint embedding methods did not obviously show better results than single-model data (such as Figures S4 & S6). In my opinion, before the joint analysis, we should carefully check the consistency between the single-model results. Joint is not always better than single (it is a general suggestion for joint analysis..)

*We appreciate this valid concern. We do believe that our joint model offers an advantage over unimodal visualizations in the specific cases mentioned by this reviewer. The joint visualization reduces the mixing of CD4+ and CD8+ T cells in the mRNA embedding, and better separates dendritic cells from CD14+ cells (previous Figure S4) and naive from memory CD4 T cells (previous Figure S6) in the ADT embedding. In addition, we confirm this visual interpretation quantitatively in Supplementary Table S2. Despite cell type specific corrections, the improvement is reflected in a global measure of separation such as the Silhouette score.*

*At the same time, we agree that it is difficult to comprehensively assess the benefit of our joint model on real data sets alone. We therefore added a simulation study in which we generated 8 synthetic data sets using parameters learned from real CITE-seq data (see description in text). Among others, the results highlight the importance of the modality weights learned by j-SNE and j-UMAP to yield consistently accurate embeddings (Supp Figs S7-S10). Concerning the specific suggestion by this reviewer, the user has control over the degree to which our method will make use of multiple modalities. Higher expected levels of noise would suggest lower values of lambda to allow non-uniform weights to counteract noisy modalities (see e.g. data set GeqN5K with 40% of cells swapped in Supplementary Figure S3). For values of lambda close to 0, our method will tend to pick the one most informative modality (Supp Fig S5) and thus behave similarly to traditional t-SNE and UMAP on unimodal data.*

**Second round of review**

**Reviewer 1**

The authors did a very thorough job addressing all reviewers' comments. I recommend acceptance.

Some very minor points in response to the authors' rebuttal:

* I said that (1-p_ij) term is approximated by 1 in UMAP, and the authors wrote that they could not confirm that in the original arXiv preprint. See page 20, "with membership strength 0" and "can be approximated by a uniform distribution" -- these phrases mean that (1-p_ij) factor is simply ignored. However, this is not important for this paper so it's okay not to mention.

* Regarding UMAP vs FIt-SNE runtime -- of course it is fine to keep the remark that FIt-SNE has linear complexity, in fact I think it'd be helpful to keep it (currently the authors took it out). What I meant is that "theoretical" complexity of both FIt-SNE and UMAP is O(n), in the sense that the optimization loop goes over n. However, empirically things slow down due to kNN search and other overhead. I'd suggest to formulate it such that it's clear for the reader that UMAP's and FIt-SNE's runtimes are both approximately linear...

* In the rebuttal, the authors write that later iterations of jNSE are faster than the 1st one because kNN graph does not need to be recomputed -- I think it's worth saying this explicitly in the text, at least somewhere in the Suppl Methods, in the Implementation section.
